# Claudin-1 decrease impacts epidermal barrier function in atopic dermatitis lesions dose-dependently

**DOI:** 10.1038/s41598-020-58718-9

**Published:** 2020-02-06

**Authors:** Sophia Bergmann, Barbara von Buenau, Sabine Vidal-y-Sy, Marek Haftek, Ewa Wladykowski, Pia Houdek, Susanne Lezius, Hélène Duplan, Katja Bäsler, Stephan Dähnhardt-Pfeiffer, Christian Gorzelanny, Stefan W. Schneider, Elke Rodriguez, Dora Stölzl, Stephan Weidinger, Johanna M. Brandner

**Affiliations:** 1 Department of Dermatology and Venerology, University Hospital Hamburg-Eppendorf, Hamburg, Germany; 2 CNRS UMR5305 LBTI and University Lyon1, Lyon, France; 3 Institute for Medical Biometry and Epidemiology, University Hospital Hamburg-Eppendorf, Hamburg, Germany; 4 Pierre Fabre Dermocosmétique, Pierre Fabre Research and Development Center Toulouse, Toulouse, France; 5 Microscopy Services Dähnhardt GmbH, Flintbek, Germany; 6 Department of Dermatology, Venerology and Allergy, University Hospital Schleswig-Holstein, Campus Kiel, Kiel, Germany

**Keywords:** Molecular biology, Skin diseases

## Abstract

The transmembrane protein claudin-1 is a major component of epidermal tight junctions (TJs), which create a dynamic paracellular barrier in the epidermis. Claudin-1 downregulation has been linked to atopic dermatitis (AD) pathogenesis but variable levels of claudin-1 have also been observed in healthy skin. To elucidate the impact of different levels of claudin-1 in healthy and diseased skin we determined claudin-1 levels in AD patients and controls and correlated them to TJ and skin barrier function. We observed a strikingly broad range of claudin-1 levels with stable TJ and overall skin barrier function in healthy and non-lesional skin. However, a significant decrease in TJ barrier function was detected in lesional AD skin where claudin-1 levels were further reduced. Investigations on reconstructed human epidermis expressing different levels of claudin-1 revealed that claudin-1 levels correlated with inside-out and outside-in barrier function, with a higher coherence for smaller molecular tracers. Claudin-1 decrease induced keratinocyte-autonomous IL-1β expression and fostered inflammatory epidermal responses to non-pathogenic *Staphylococci*. In conclusion, claudin-1 decrease beyond a threshold level results in TJ and epidermal barrier function impairment and induces inflammation in human epidermis. Increasing claudin-1 levels might improve barrier function and decrease inflammation and therefore be a target for AD treatment.

## Introduction

Tight junctions (TJs) are an important component of the complex epidermal barrier system. They are localized in the *stratum granulosum* (SG) of the epidermis and provide mechanical barrier function to ions and solutes of different molecular sizes^1–4^.

The transmembrane protein claudin-1 (Cldn-1) is a major component of TJs^5^. It is also found outside of TJs in basal and suprabasal layers of the epidermis^2,5^. Mice with a complete Cldn-1 knock-out (KO) die at the first day of birth due to increased transepidermal water loss (TEWL)^5^. They develop TJs leaky to a molecular tracer (Biotin-556)^5^, and a highly water permeable *stratum corneum* (SC)^6^. Human subjects lacking Cldn-1 suffer from the Neonatal Ichthyosis-Sclerosing Cholangitis (NISCH) syndrome which includes an ichthyosiform skin phenotype^7^.

An archetypical disease of epidermal barrier dysfunction is atopic dermatitis (AD)^8^. Cldn-1 single nucleotide polymorphisms were linked to AD in some cohorts^9–11^, but not in others^11,12^. Using immunostaining-intensity measurements and western blot analyses, reduced Cldn-1 levels were found in lesional AD skin^13–16^. For non-lesional skin, divergent observations were described. Some authors found decreased mRNA and immunointensity levels^10^, while others observed no alteration of Cldn-1 immunointensity and western-blot levels^14,16^. Also in healthy skin, variability of Cldn-1 levels was detected^10,13–15^, but was not a subject of further investigation and discussion yet. Recently, it was shown that decreased Cldn-1 levels can be correlated dose-dependently to an increased number of macrophages in human AD skin and to several features of inflammation in mice^15^. The correlation of Cldn-1 levels to skin and/or TJ barrier function in human has not been investigated so far.

Concerning barrier function, elevated TEWL (e.g.^14,17^), increased outside-in permeation of molecular tracers^10,18,19^, and decreased electrical resistance^10^ were described in AD. Alterations are often much more pronounced in lesional compared to non-lesional skin. However, these parameters describe the overall (mechanical) epidermal barrier which is composed of TJ and SC barrier. A comparative investigation of specifically TJ barrier function in lesional and non-lesional AD skin is missing up to now.

To gain further insight into the role of TJs and Cldn-1 in epidermal dysfunction in AD, we determined Cldn-1 levels in healthy as well as lesional and non-lesional skin from AD patients and correlated the results to TJ barrier function identified by inside-out permeation of Biotin-556 (“Biotinylation assay”^2,4,5^) and overall epidermal barrier function measured by TEWL. Furthermore, we established *in-vitro* 3D models of reconstructed human epidermis (RHE) using normal human epidermal keratinocytes which expressed different levels of Cldn-1 due to Cldn-1 knock-down (KD). In these models we correlated Cldn-1 levels with transepithelial electrical resistance (TER), TJ inside-out barrier function to molecules of different sizes, and outside-in overall epidermal barrier function to molecular tracers, as well as the induction of inflammatory response (IL-1β expression). Because AD is often associated with *staphylococcal* colonialization/infection of the skin, we also challenged these models by non-pathogenic and pathogenic *Staphylococci* and investigated their impact on barrier function and induction of inflammatory response in interplay with Cldn-1 KD.

## Results

### Varying Cldn-1 levels in healthy skin

**Figure 1:**
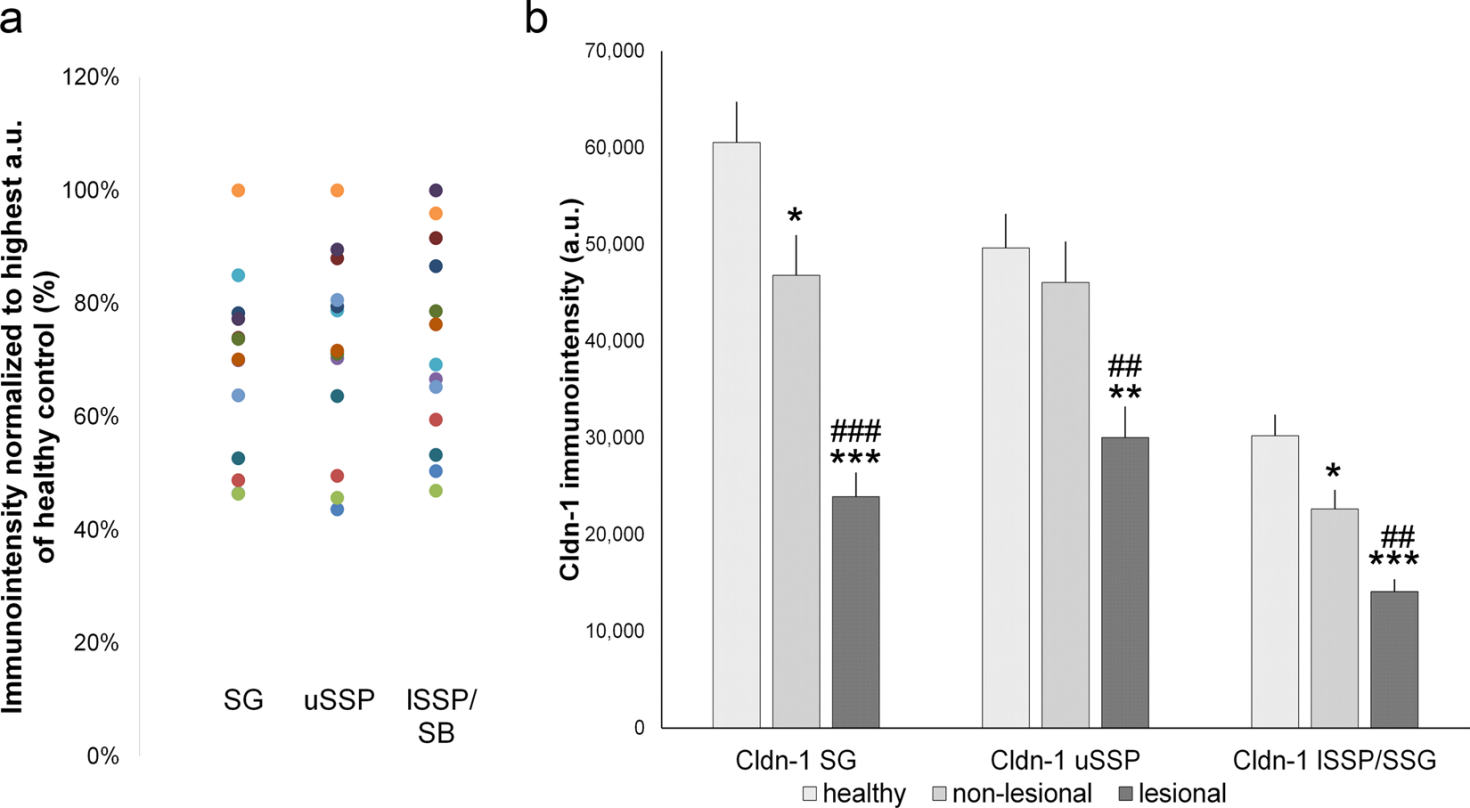
Cldn-1 immunointensity levels in a new Northern German cohort. (**a**) Cldn-1 immunostaining-intensity in *stratum granulosum* (SG), upper *stratum spinosum* (uSSP) and lower SSP/*stratum basale* (lSSP/SB) of healthy skin normalized to the sample with highest immunointensity in the respective layer. Dots of the same color belong to the same individual. (**b**) Immunointensity of Cldn-1 in SG, uSSP and lSSP/SB in healthy, non-lesional and lesional AD skin. Mean + SEM. n = 13 per group. *:significant compared to healthy control; # significant compared to non-lesional skin. a.u. arbitrary units.

We investigated Cldn-1 immunostaining-intensity in healthy as well as lesional and non-lesional AD skin in a cohort from Northern Germany. To get more detailed information on Cldn-1 in different epidermal layers, where it most likely fulfils different functions, we investigated immunointensity of Cldn-1 in the SG, upper *Stratum spinosum* (uSSP), and lower epidermal layers (lower SSP (lSSP)/*stratum basale* (SB)) (see Supplementary Fig. S1) separately. We observed varying Cldn-1 levels in all layers of healthy epidermis. In SG, Cldn-1 immunointensity was ranging from 46% to 100% of maximum staining intensity, in uSSP from 44% to 100% and in lSSP/SB from 47% to 100% (Fig. 1a).

### Significantly decreased expression of Cldn-1 in non-lesional and lesional AD skin

When comparing healthy to non-lesional and lesional skin in this new cohort we observed significantly decreased immunointensity of Cldn-1 in lesional AD skin compared to healthy skin and non-lesional AD skin in all three layers (Fig. 1b). In addition, there was a significant, but less pronounced, decrease in non-lesional AD skin compared to healthy controls in SG and lSSP/SB but not in uSSP (Fig. 1b).

Furthermore, we investigated claudin-4 (Cldn-4) and occludin (Ocln) and observed up-regulation of both proteins in non-lesional skin and downregulation (Cldn-4 in SG) as well as upregulation (Cldn-4 in uSSP and mid SSP (mSSP), Ocln in all layers) in lesional skin (see Supplementary Figs. S1–S3).

### Stable Biotin-556 TJ barrier in healthy and non-lesional skin within Cldn-1 levels of 50–100%

**Figure 2:**
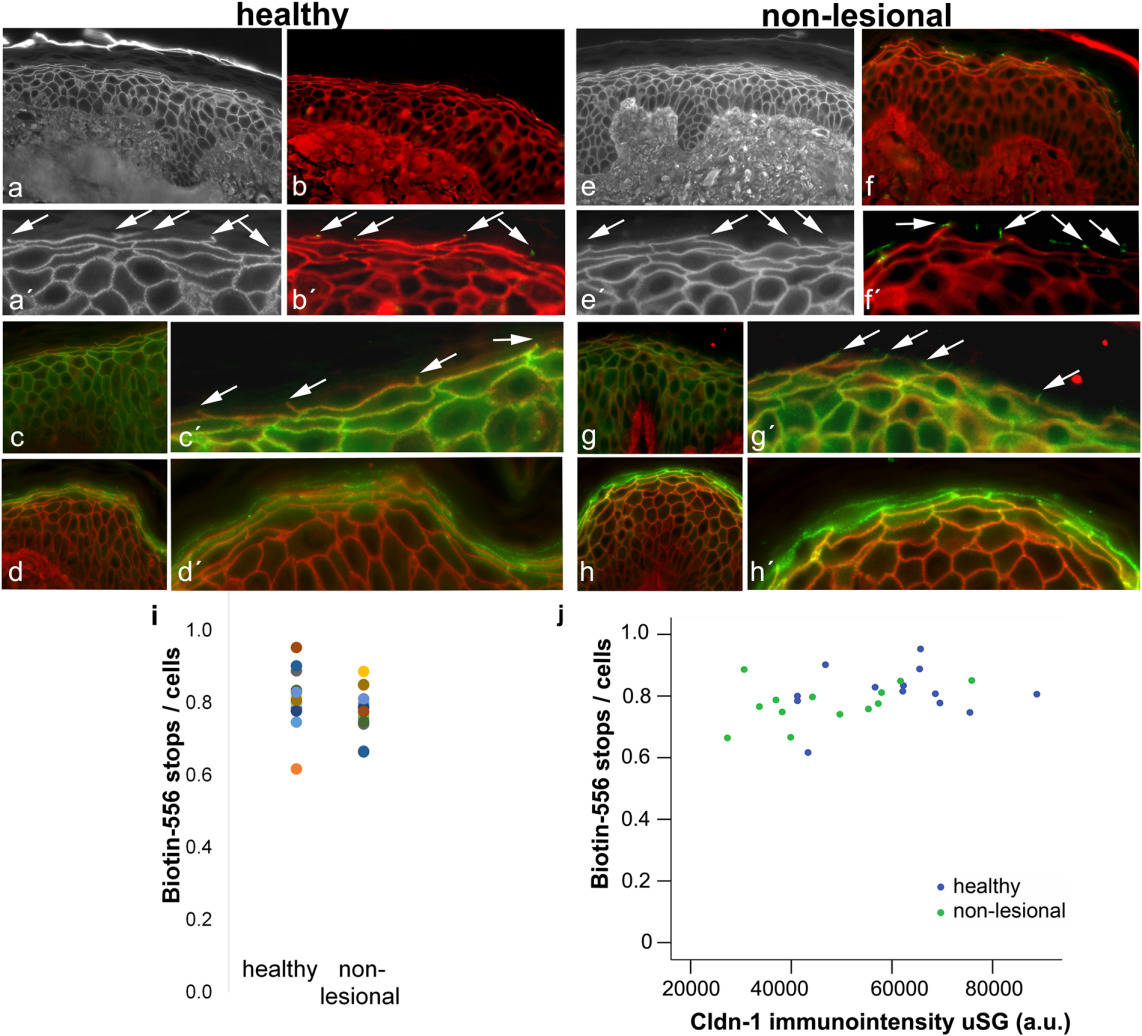
TJ barrier in healthy and AD non-lesional skin and its correlation to Cldn-1 levels. (**a**,a′,**e**,e′) Examples for Biotin-556-TJ barrier assays in healthy (**a**,a′) and non-lesional AD (**e**,e′) skin. (**b**,b′,**f**,f′) Double-staining of Biotin-556 (red) and occludin (green), (**c**,c′,**g**,g′) of Biotin-556 (red) and Cldn-1 (green), and (**d**,d′,**h**,h′) of Biotin-556 (red) and Cldn-4 (green) in healthy (**b**,b′,**c**,c′,**d**,d′) and non-lesional (**f**,f′,**g**,g′,**h**,h′) skin. (a′,b′,c′,d′,e′,f′,g′,h′) are magnifications of (**a–h**). Arrows denote “tracer-stops”. (**i**) Number of tracer stops per number of cells in the underlying layer in healthy and AD non-lesional skin. Each dot represents a proband/patient (n = 13 per group). (**j**) Correlation of Biotin-556 tracer stops/cells with Cldn-1 immunointensity in SG in healthy and non-lesional AD skin. Blue dots: healthy, green dots: non-lesional.

To test TJ inside-out barrier we dermally injected Biotin-556 which is known to be stopped at functional TJs^4,5^. Several stopping points of the tracer (“tracer-stops”), could be distinctly seen in the SG of healthy and non-lesional skin (Fig. 2a,a′,e,e′). These stops clearly colocalize with occludin (Fig. 2b,b′,f,f′) which has been used before as a marker for TJs^5,20^. They also colocalize with claudin-1 (Fig. 2c,c′,g,g′) and claudin-4 (Fig. 2d,d′,h,h′). The latter is often also found in the cells above the Biotin-stops. The number of Biotin-stops per number of cells in the underlying layer varied between 0.62/cells to 0.95/cells in healthy skin and 0.67/cells to 0.89/cells in non-lesional skin without statistical significant differences between healthy and non-lesional skin (Fig. 2i). The number of tracer stops was largely constant within a wide range of SG-Cldn-1 levels (< approx. 45,000 arbitrary units (a.u.), i.e. approx. 50–100% of maximum Cldn-1 immunointensity, Fig. 2j). Only at lowest levels there was a slight decline (Fig. 2j). Similar results were found for Cldn-1 levels in uSSP and lSSP/SB (Supplementary Fig. S4).

### Increased TJ and skin permeability in AD lesions in a Cldn-1-level correlating manner

**Figure 3:**
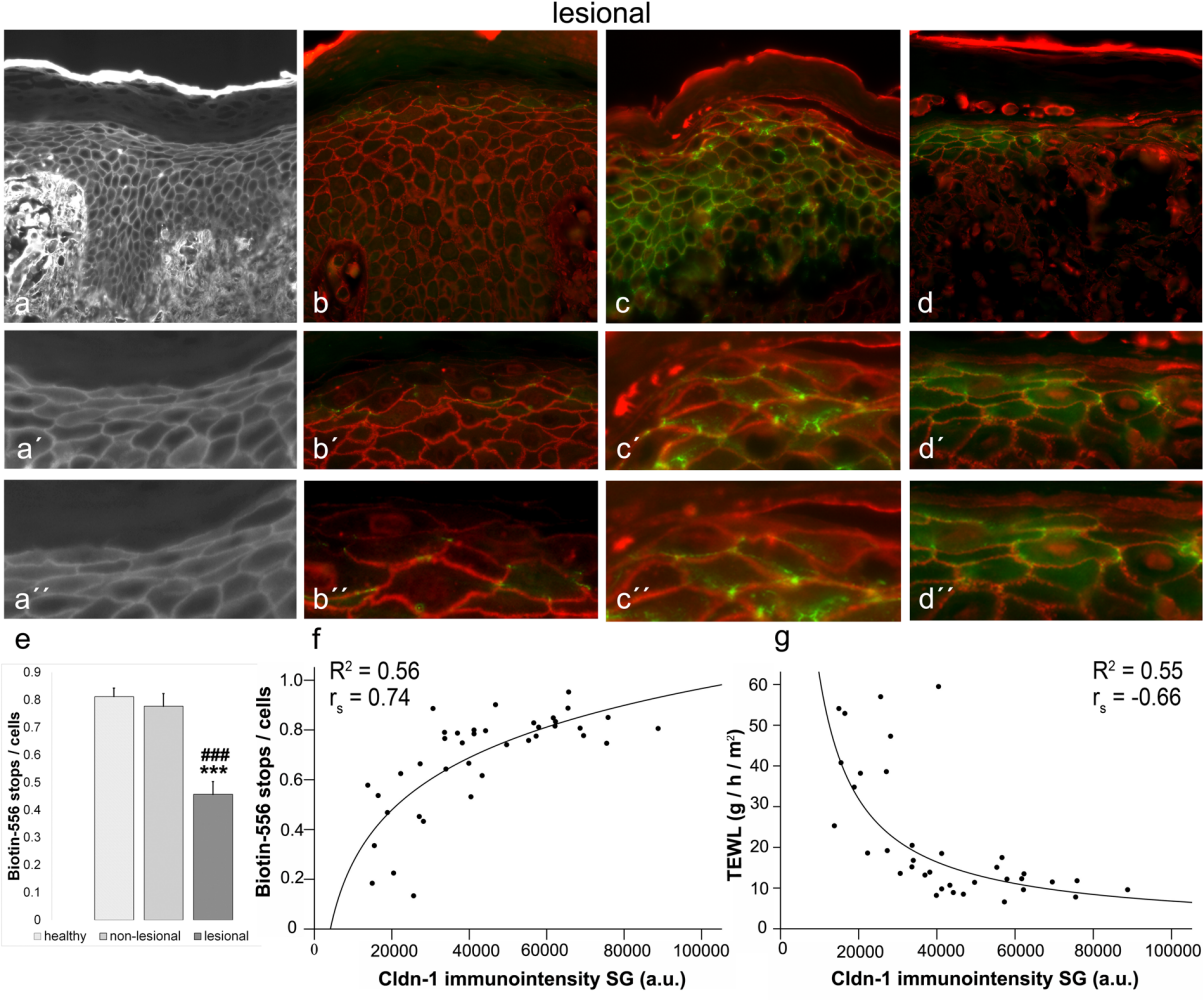
TJ barrier in lesional AD skin and its correlation to Cldn-1 levels. (**a**,a′,a′′) Example for Biotin-556-TJ barrier assay in lesional AD skin. (**b**,b′,b′′) Double-staining of Biotin-556 (red) and occludin (green), (**c**,c′,c′′) of Biotin-556 (red) and Cldn-1 (green), and (**d**,d′,d′′) of Biotin-556 (red) and Cldn-4 (green) in lesional AD skin. (a′,b′,c′,d′) are magnifications of (**a**–**d**). (a′′,b′′,c′′,d′′) are magnifications of (a′,b′,c′,d′). (e) Quantitative evaluation of Biotin-556 tracer stops/cells in healthy, non-lesional and lesional AD skin. (**f**,**g**) Correlation of Cldn-1 immunointensity in SG to Biotin-556 stops/cells (**f**) and TEWL (**g**). (**e**) Mean + SEM. n = 13 per group. *Significant compared to healthy control; #significant compared to non-lesional skin. a.u. arbitrary units. Bars: 50 µm.

In AD lesional skin, stainings often did not show the classical “tracer-stops” (Fig. 3a,a′,a′′), but Biotin-556 penetrated through occludin (Fig. 3b,b′,b′′), Cldn-1 (Fig. 3c,c′c′′) and Cldn-4 (Fig. 3d,d′,d′′) positive sites. The number of tracer-stops was significantly reduced compared to healthy and non-lesional skin (Fig. 3e). Combining the results of Biotin-556-TJ barrier function in correlation to Cldn-1 levels in SG for healthy, non-lesional and lesional AD skin, led to the observation of an overall correlation with good fit to a logarithmic curve (Fig. 3f, Coefficient of determination (R^2^) = 0.56, Spearman′s correlation coefficient (r_S_) = 0.74, p < 0.01). For uSSP and lSSP/SB only correlations with low R^2^-values (R^2^ = 0.31, r_S_ = 0.60 and 0.66 respectively; p < 0.01) were found (Supplementary Fig. S4). Thus we further focused on Cldn-1 in SG, also supported by the fact that this is the area where the TJ barrier is localized.

When correlating Cldn-1 levels in the SG with TEWL as overall epidermal inside-out barrier parameter, we observed a good fit to a curve of a power function (Fig. 3g; R^2^ = 0.55, r_S_ = −0.66). TEWL was quite stable above 40,000 a.u. of Cldn-1 immunointensity (approx. 45% of maximum); at lower expression levels permeability raised.

### Cldn-1 KD diminishes inside-out and outside-in barriers at lower Cldn-1 levels in RHE

Because there are many influences in AD skin in addition to Cldn-1 levels, we wanted to further investigate the impact of Cldn-1 levels on human TJ and overall epidermal barrier function in more detail in a system similar to human epidermis but with identical influencing parameters except for Cldn-1 levels. Therefore we built RHE with keratinocytes expressing different Cldn-1 levels due to KD (see methods).

We investigated RHE at two different time points, day 4 for a time point reflecting development/regeneration of the barrier and day 8 for mature RHE^21,22^ to get a broader insight on the effect of Cldn-1 in the epidermis. At both time points functional, Biotin-556-stopping, TJs could be observed at occludin- (Supplementary Fig. S6-8) and claudin-1-positive sites (Supplementary Figs. S7 and S8), however, number of TJ “stops” increased from day 4 (4.1 ± 0.8 per visual field (n = 6)) to day 8 (12.5 ± 1.2 per visual field (n = 7). For a general description of morphology, differentiation and barrier function of the RHE see Supplementary data including Supplementary Figs. S5 and S6.

**Figure 4:**
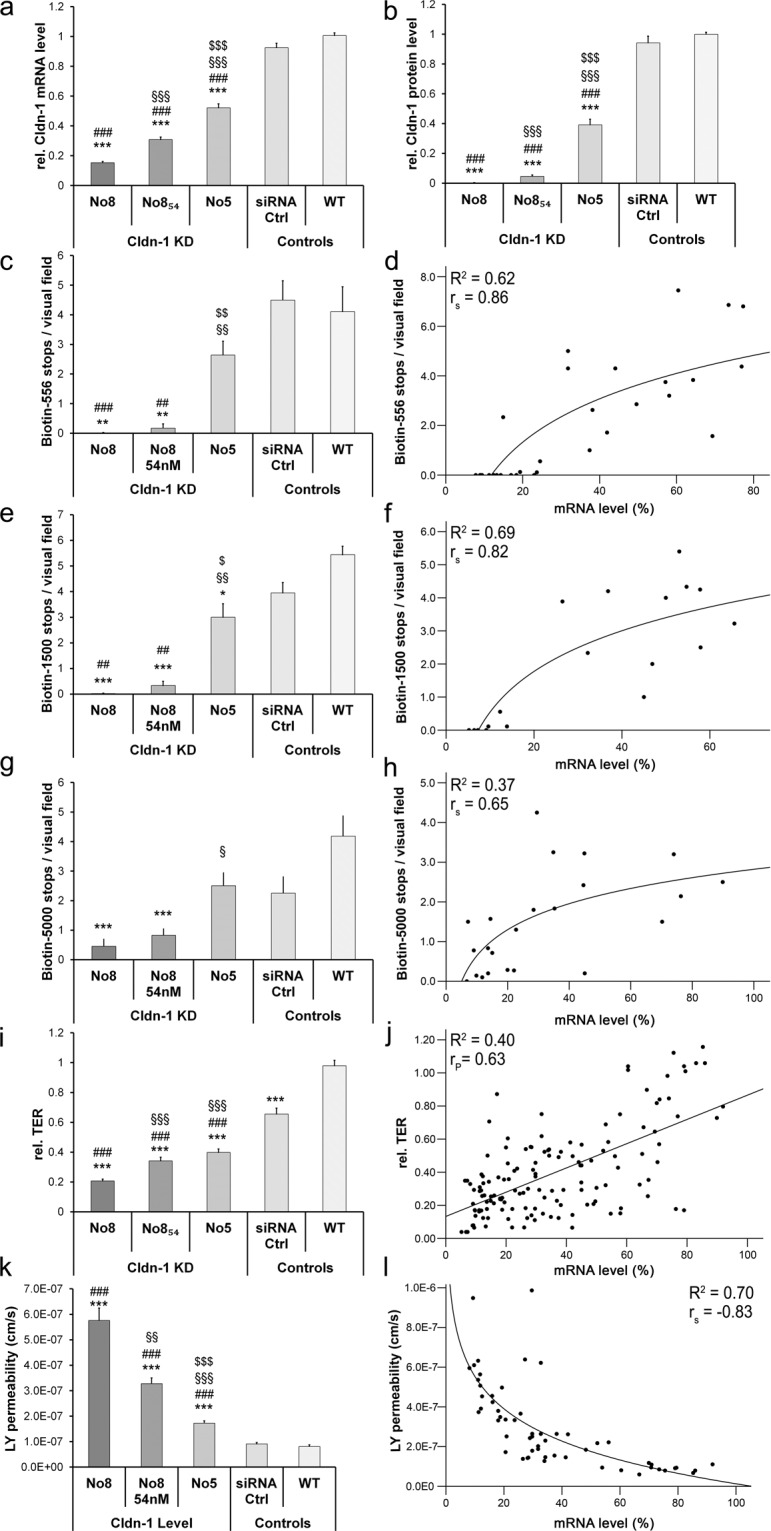
Influence of different Cldn-1 levels on TJ barrier and epidermal barrier function in RHE at day 4. (**a**,**b**) mRNA (**a**) and protein (**b**) levels achieved by the different Cldn-1 knock-down (KD)-siRNA treatments as well as controls normalized to the individual wt control per experiment (**a**: n = 10 different donors, **b**: n = 12 different donors). (**c**,**e**,**g**,**i**,**k**) Number of Biotin-556 (**c**), Biotin-1500 (**e**), and Biotin-5000 (**g**) stops/visual field, as well as TER (**i**) and Lucifer Yellow (LY) permeability (**k**) achieved by the different Cldn-1 KD-siRNA treatments as well as controls (n = 6, 4, 3, 12, 4 different donors). WT: wild type. (**d**,**f**,**h**,**j**,**l**) direct correlations of Biotin-556 (**d**), Biotin-1500 (**f**), Biotin-5000 (**h**), TER (**j**) and LY permeability (**l**) to mRNA levels normalized to the wt control with highest Cldn-1 level of all experiments. Significances: *to wt, ^#^to siRNA ctrl, ^$^to No 8_54 nM_ and ^§^ to No. 8.

We observed significant reductions of Cldn-1 mRNA and protein to different levels in RHE treated with siRNA No. 8, siRNA No. 8_54nM_ and siRNA No. 5 at day 4 and day 8 (Fig. 4a,b, Supplementary Fig. S9a,b). The strongest effect was achieved by siRNA No. 8 followed by siRNA No. 8_54nM_ and siRNA No. 5. KD of Cldn-1 did not result in peculiar morphological changes or alteration of proliferation in the RHE (see Supplementary data including Supplementary Figs. S5-8).

Concerning other TJ proteins, there was no significant impact of Cldn-1 KD on expression of Ocln, zonula occludens protein 1 (ZO-1) and junctional adhesion molecule A (JAM-A) at day 4 and day 8 (Supplementary Table S3, for Ocln staining see also Supplementary Figs. S7 and S8), while there was a significant but not dose-dependent downregulation of Cldn-4 at day 4 (reduction to 0.65 ± 0.03 with siRNA No. 5 and to 0.64 ± 0.05 with siRNA No. 8, p < 0.001). At day 8 there was no influence on Cldn-4 mRNA.

For barrier function analyses, we investigated inside-out TJ barrier function by using 3 different sizes of biotin-tracers (Biotin-556, Biotin-1500 and Biotin-5000), outside-in overall epidermal barrier by using the molecular tracer Lucifer yellow (LY) (457 Da) and overall epidermal ion barrier by TER measurement.

At day 4, Cldn-1 KD impaired the barrier function to all three biotin-tracers. The effect was stronger in siRNA treatment groups with more pronounced Cldn-1 KD (Fig. 4c,e,g). For Biotin-5000 moderate KD as derived with siRNA No. 5 did not have an impact on permeability. When directly correlating Cldn-1 mRNA levels and tracer-stops we observed a good fit to a logarithmic curve for Biotin-556 (Fig. 4d; R^2^ = 0.62, r_S_ = 0.86, p < 0.01) and Biotin-1500 (Fig. 4f; R^2^ = 0.69, r_S_ = 0.82; p < 0.01). For Biotin-5000 only a R^2^ value of 0.37 (r_S_ = 0.65; p < 0.01) was achieved (Fig. 4h). Even though barrier function to the Biotins was impaired in Cldn-1 KD preparations, there was still a dot-like staining of Ocln, however without tracer stop (Supplementary Fig. S7), similar to what was found in Cldn-1 knock-out mice^5^.

Also TER was significantly and dose-dependently diminished in our Cldn-1 KD experiments with the different siRNA preparations at day 4 (Fig. 4i). Direct correlation between Cldn-1 levels and TER exhibited a significant linear correlation, but with only moderate R^2^ value (R^2^ = 0.4; Pearson′s correlation (r_P_) = 0.63; p < 0.01; Fig. 4j). In addition, we observed significant and dose-dependent diminution of the outside-in barrier function to LY (Fig. 4k). Correlation of Cldn-1 level and LY permeability fitted to a curve describing a power function (Fig. 4l; R^2^ = 0.7, r_S_ = −0.83; p < 0.01).

We also tested the general influence of Cldn-1 KD on inside-out barrier function to ions by using lanthanum in electron microscopy. We observed a clear influence of Cldn-1 KD on TJ barrier function permitting lanthanum penetration into the SG/SC interface, above the usual level of the tracer-stop in SG2 (Supplementary Fig. S10).

Also at day 8 there was a significant impairment of the barrier function to all three biotin-preparations after strong Cldn-1 KD. However, moderate KD as seen with siRNA No. 5 did not have an effect (Supplementary Fig. S9c,e,g), and treatment with siRNA No. 8_58nM_ - which resulted in intermediate Cldn-1 KD - had less effect than at day 4. In general, correlation of barrier read-out parameters with Cldn-1 mRNA showed much lower R^2^ values than at day 4 (for detailed description see supplementary data). For Biotin-556 and Biotin-1500 a quite stable barrier function was observed at Cldn-1 mRNA levels >50–60%, below this value a clear decrease was seen (Supplementary Fig. S9d,f). The same was true for LY permeation (Supplementary Fig. S9k,l)

### No additional effect of *Staphylococcal* infection on barrier function in Cldn-1 KD RHE

Patients suffering from AD often have additional complications by skin infections. In order to unravel interaction of bacteria and Cldn-1 KD concerning barrier impairment we infected our RHE models 48 h before analysis with pathogenic S. *aureus* and non-pathogenic S. *carnosus*. S. *aureus* increased permeability in wild type (wt) and control siRNA-treated RHE  while this was not the case for S. *carnosus*. However, there was no significant additional effect of bacteria on TER or LY permeability in RHE with Cldn-1 KD. For a detailed description of bacteria induced barrier alterations see Supplementary data including Supplementary Fig. S11.

### Cldn-1 KD impacts lipid lamellae structure and decreases filaggrin expression

To investigate whether Cldn-1 KD might result in compensatory or aggravating effects in SC morphology, we investigated SC lipid structure and proteins.

**Figure 5:**
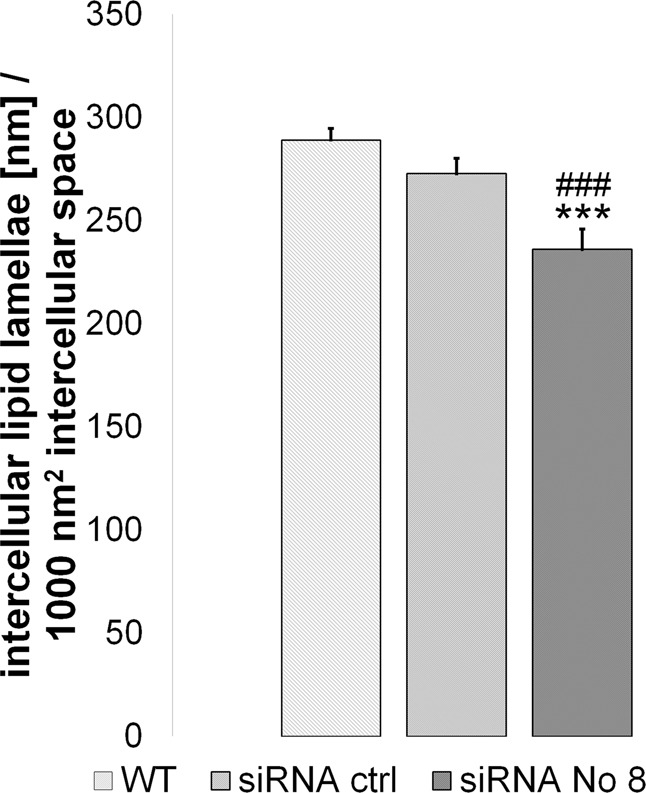
Influence of Cldn-1 KD on lipid lamellae length at day 8. Mean + SEM. n = 30 from 2 different donors *  wt compared to Cldn-1 KD achieved by siRNA No. 8. ^#^siRNA control compared to siRNA No. 8.

Normalized intercellular lipid lamellae length per 1000 nm^2^ intercellular space^23^ was significantly reduced after Cldn-1 KD at day 8 (Fig. 5).

Concerning SC proteins there was no significant influence of Cldn-1 KD on expression of involucrin, loricrin and repetin at day 8 (see Supplementary Table S4). A significant downregulation of filaggrin (Flg) mRNA was observed for the strongest KD (siRNA No. 8; downregulation to 68% ± 6%, p < 0.05 compared to wt).

### Cldn-1 KD causes early inflammatory responses

To test whether Cldn-1 level has an influence on expression of inflammatory markers in human keratinocytes, we investigated IL-1β expression in our Cldn-1 KD RHE.

**Figure 6:**
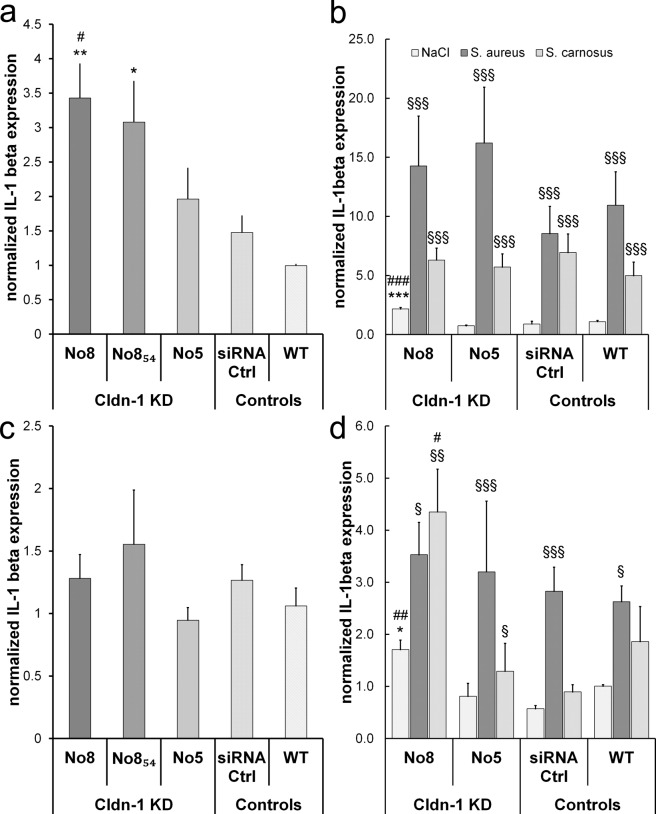
Influence of Cldn-1 KD and *staphylococcal* infection on IL-1β expression in RHE. Normalized IL-1β mRNA levels in wild type (WT) and siRNA control (siRNA Ctrl) as well as in with the different Cldn-1 knock-down (KD)-siRNA preparations-treated RHE at day 4 (**a**,**b**) and day 8 (**c**,**d**). (**a**,**c**) Influence of Cldn-1 KD; (**b**,**d**) Influence of Cldn-1 KD and bacterial infection. Mean + SEM. (**a**,**c**) *compared to wt ctrl, ^#^compared to siRNA ctrl, n = 4 (**b**,**d**) *compared to NaCl wt ctrl, ^#^compared to respective siRNA ctrl, ^§^compared to NaCl ctrl within the treatment group n = 3.

We observed a dose-dependent increase in IL-1β expression in RHE with Cldn-1 KD at day 4 (Fig. 6a). At day 8 no significant differences compared to controls were observed (Fig. 6c).

When combining Cldn-1 KD and bacterial infection with S. *carnosus* or S. *aureus* we observed that both bacteria increased IL-1β levels in wt RHE at day 4. The effect was much more pronounced for S. *aureus* (Fig. 6b: p < 0.01 for comparison between bacterial strains). The influence of S. *carnosus* at day 4 was independent from the Cldn-1 KD level. For S. *aureus* there was a stronger effect in KD RHEs by trend, but there was no dose-dependent effect. At day 8 *S*. *aureus* increased IL-1β levels significantly (Fig. 6d), but there was no additional effect in Cldn-1 KD RHE. However, S. *carnosus* which did not show a significant effect on IL-1β levels in control RHE, exhibited a moderate but significant IL-1β increasing effect in RHE with moderate Cldn-1 KD (siRNA No. 5) and a strong increasing effect in RHE with strong Cldn-1 KD (siRNA No. 8) (Fig. 6d).

We also investigated the effect of Cldn-1 KD and bacterial infection on toll-like-receptors TLR1, TLR2, and TLR3 as well as NOD2 at day 4 and day 8. We did not observe any significant effect of Cldn-1 KD in infected RHE on TLR/NOD2-expression, neither at day 4 nor at day 8, even though we observed an increase of TLR/NOD2 due to *S*. *aureus* inoculation (TLR2 in wt controls at day 4 inoculated with *S*. *aureus*: 4.0 fold ± 0.7, p < 0.05 compared to uninfected controls, n = 3 independent donors; inoculated with *S*. *carnosus* 1.6 fold ± 0.3, p = 0.05 compared to uninfected controls, n = 3 independent donors; NOD2 in wt controls at day 4 inoculated with *S*. *aureus*: 2.0 fold ± 0.9, n = 3 indepenent donors; inoculated with *S*. *carnosus* 1.3 fold ± 0.2, n = 3 independent donors).

## Discussion

Cldn-1 was shown before to play a dose-dependent role in cultured keratinocyte barrier function and epidermal inflammation in mice^15^. However, data correlating different Cldn-1 levels and epidermal barrier function in Man were missing so far. By using healthy, non-lesional and lesional AD skin with different Cldn-1 levels as well as RHE with different Cldn-1 levels induced by siRNA-mediated KD, we show here that there is a dose-dependent correlation in human epidermis between Cldn-1 levels and both, TJ barrier and overall epidermal barrier function. In addition, we show on the one hand that there is a keratinocyte-autonomous influence of Cldn-1 KD on inflammatory response, and on the other hand that Cldn-1 KD also fosters inflammatory effects of non-pathogenic *Staphylococci*.

We observed, similar to a Northern American cohort^10^, a decrease of Cldn-1 expression in non-lesional skin in our Northern German cohort. Specifically, this decrease could be identified in the SG and in the lSSP/SB, but not in the uSSP, revealing an epidermal layer-dependent effect. This is of importance, because function of Cldn-1 is supposedly different at diverse epidermal levels. In SG, Cldn-1 is associated with TJs and permeability barrier function^2,4,24^. In uSSP it may be mostly involved in epidermal differentiation and in the lower epidermis in the proliferation/differentiation balance^10,14^.

Of note, despite the decrease of Cldn-1 levels in non-lesional skin, we did not observe impaired TJ barrier function (Biotin-556) or overall epidermal barrier function (TEWL). In addition, we demonstrate here that also in healthy skin there was a broad range of Cldn-1 levels, but impaired TJ and overall epidermal barrier function could only be observed at lowest levels. In lesional AD skin, where there was a further significant decrease in Cldn-1 levels (this manuscript and^13,14,16^), we observed a clear decrease in TJ barrier function and a clear increase of TEWL (see also^14,17^ for increased TEWL in AD lesions). Altogether, we observed that below a certain Cldn-1 level of approx. 50% there was a clear decrease in barrier function of the skin. This suggests that skin can compensate for Cldn-1 decrease up to a certain level but that there is a minimal threshold level of Cldn-1. Remarkably, DeBenedetto *et al*. who described a more pronounced decrease of Cldn-1 in non-lesional skin to approx. 45% of healthy controls (versus approx. 75% in our study) in a Northern American cohort, observed decreased electrical resistance and increased outside-in albumin permeability in non-lesional skin^10^. Yoshida *et al*.^25^ performed the Biotin-556 TJ assay in samples of AD erythematous skin specifically chosen because of Cldn-1 levels similar to the healthy skin. In line with our results they did not observe impaired TJ barrier function in these samples.

This suggests that, indeed, Cldn-1 decrease and no other parameters of AD are primarily linked to decreased TJ barrier function. To support this hypothesis we decided to use an experimental system where only Cldn-1 levels were manipulated. Therefore we knocked-down Cldn-1 to different levels in RHE.

We observed a correlation between Cldn-1 and Biotin-556 in RHE which fitted - similar to human skin - to a logarithmic curve. This could also be seen for Biotin-1500. For Biotin-5000 the correlation was less pronounced and barrier function to this molecule was less dependent on Cldn-1 levels. This is in line with a size-dependent impairment of TJs shown after allergic inflammation^3^. For TER, correlation was linear, suggesting different influence of Cldn-1 on barrier to ions and barrier to molecular tracers. In addition, TER exhibited a lower correlation coefficient compared to biotins, hinting for the influence of SC on the ion barrier. The influence of the SC also likely explains the lower correlation coefficients of LY and TER at day 8 compared to day 4 because these parameters do not only reflect TJ barrier function but also SC barrier function and at day 8 more layers of SC are present. In submerged cultured mouse keratinocytes, which do not form a SC, a very good correlation between Cldn-1 levels and TER was described^15^.

However, one has to keep in mind that also SC barrier function is influenced by Cldn-1 KD. We show here that Cldn-1 KD resulted in alteration of lipid lamellae structure and filaggrin mRNA expression. It was shown before in mice that Cldn-1 KO altered SC lipid composition and filaggrin processing^6^. Thus, even though the increased number of SC layers at day 8 compared to day 4 influence barrier function, the additional SC layers cannot completely compensate for Cldn-1 KD.

Unexpectedly, also for inside-out TJ barrier to the biotins, the correlation to Cldn-1 levels was lower at day 8 than at day 4. This might reflect compensatory mechanisms within the TJ composition. Indeed, at day 8 Cldn-4 levels were higher than at day 4 in Cldn-1 KD cells.

Correlation coefficients between Cldn-1 levels and Biotin-556 were slightly higher in RHE at day 4 and lower at day 8 than in the human skin samples, demonstrating the importance of usage of different models to frame the *in-vivo* situation. Interestingly, at day 8 the correlation graphs showed that there was a quite constant barrier function to Biotin-556 and Biotin-1500 as well as LY in RHE with mRNA levels above 50–60%. At lower Cldn-1 levels, the barrier function decreased, which is similar to the situation observed *in-vivo*.

Because it was shown in mice that loss of Cldn-1 increased the expression of the pro-inflammatory cytokine IL-1β^15^ dose-dependently, we tested whether the loss of Cldn-1 resulted in a change of this proinflammatory marker in human keratinocytes as well and whether it is therefore a system-independent, keratinocyte-autonomous effect. Indeed, RHE with Cldn-1 KD showed a dose-dependent upregulation of IL-1β. This clearly indicates that Cldn-1 down-regulation can be the starting point of inflammatory response. When combining Cldn-1 KD and *Staphylococci* we observed that the non-pathogenic strain S. *carnosus*, which did not significantly induce IL-1β at day 8 in controls, exhibited moderate induction of IL-1β in the RHE with moderate (No. 5) and strong induction in RHE with strong barrier impairment (No. 8) due to Cldn-1 KD. IL-1β leads to infiltration of inflammatory cells^26^ and the S. *carnosus*-induced increase of IL-1β in Cldn-1 reduced cells may thus add to the vicious circle of AD. In AD lesional skin a dose-dependent correlation between Cldn-1 and macrophages was described^15^. For S. *aureus*, which is per se a strong inducer of IL-1β^27^, there was no significant additional effect, but a trend at day 4. Because uptake of allergens and haptens is a strong inducer of AD and it was shown in mice that hapten-induced dermatitis results in decreased Cldn-1 levels and opening of TJs^3^ it would be interesting to test the interaction of allergens/haptens and different Cldn-1 levels in future in human keratinocyte models.

In conclusion this study provides first evidence that defined Cldn-1 levels are important for TJ and epidermal barrier function in human skin. Strong downregulation, as seen in AD lesions, resulted in diminished inside-out and outside-in barrier function. However, there is a wide range of Cldn-1 levels between approx. 50 and 100% which can be compensated and thus do not lead to barrier impairment. Nevertheless, Cldn-1 downregulation also resulted in an induction of proinflammatory IL-1β cytokine expression and fostered occurrence of inflammation in the presence of *Staphylococci*, even those reputed non-pathogenic. Thus, increase of Cldn-1 may be a promising target for AD therapy.

## Methods

### Human tissues, antibodies, siRNAs, primers, bacterial strains

Samples from 13 adult patients with active AD (4 male, 9 female, mean age 32 years (19–61 years), mean SCORAD: 38.2) and 13 healthy controls (4 male, 9 female, mean age 33 years (19–61 years)) without personal or familial history of allergic and chronic-inflammatory skin diseases were collected at the Christian Albrechts University (CAU) Kiel and the University Hospital Hamburg-Eppendorf. Informed written consent was obtained from all subjects under a protocol approved by the local ethics board at the University Hospital Schleswig-Holstein, Campus Kiel, Germany (reference: A100/12) and the Aerztekammer Hamburg (PV4724, PV4400). AD was diagnosed on the basis of a skin examination by experienced dermatologists using the American Academy of Dermatology diagnostic criteria^28^. Exclusion criteria were presence of any other chronic skin disease, systemic treatment with immune-efficient medication ever, and topical treatment at sites of skin biopsies within one week prior to material sampling. From all participants, 9 ml of blood (collected into EDTA) as well as 5 mm skin punch biopsies from the flexural aspects of the extremities were obtained. From control individuals, a single biopsy was taken, while pairs of biopsies were taken intra-lesionally and from clinically normal skin of similar localization (at least 50 mm from active lesions) in AD patients. Two patients and one healthy control carried a single FLG mutation (for genetic analyses see Supplementary data). Human tissue for cultivation of primary keratinocytes was obtained anonymously from male donors <5 years (approved by the ethics committee of the Aerztekammer Hamburg (WF-61/12)). All investigations were conducted according to the principles expressed in the Declaration of Helsinki.

Antibodies and dilutions are listed in Supplementary Table S1, FAM-dye-labelled real-time PCR TaqMan MGB probes in Table S2 in Supplementary data. siRNAs for human Cldn1 (Hs_CLDN1_5 (No. 5): SI03206336, Hs_CLDN1_8 (No. 8): SI04279114) and AllStars Negative control (siRNA ctrl; SI03650318) were purchased from QIAGEN (Hilden, Germany).

*Staphylococcus aureus* strain SH1000^29^ and *S*. *carnosus* strain TM300^30^ were used.

### Cell culture

Primary human keratinocytes were isolated from foreskin as described before^31^, and cultured in serum-free EpiLife medium (Life Technologies, Carlsbad, USA).

### siRNA experiments

Confluent cells in passage 3 were trypsinized and resuspended in EpiLife medium with 1.5 mM CaCl_2_. To gain different levels of Cldn-1, cells were then transfected by using HiPerFect Transfection reagent (Qiagen, Hilden, Germany) at a cell density of 4 × 10^5^/mL with a final siRNA concentration of 80 nM (later called siRNA No. 8) or 54 nM (later called siRNA No. 8_54nM_) of siRNA No. 8. In addition, 80 nM siRNA No. 5 (later called siRNA No. 5) which shows less KD efficiency was used to achieve additional KD levels and to exclude off-target effects. 80 nM AllStars Negative Control siRNA was used as control. Transfected cells were directly used to build RHEs.

### Reconstructed human epidermis (RHE)

2 × 10^5^ keratinocytes were seeded in 500 μL EpiLife medium (1.5 mM CaCl_2_) onto Millicell cell culture inserts (0.4 μm, 12 mm diameter, Merck Millipore, Tullagreen, Ireland) and RHE were built as described before^21^.

### TER measurement

TER was measured by using a voltohmmeter (World Precision Instruments, Sarasota, USA) and TER values were corrected by subtracting a blank value (insert without cells). Relative TER values were calculated by normalization to the respective untreated control.

### Lucifer yellow (LY) permeability assay

Permeability of RHE for the tracer LY (Sigma-Aldrich, Munich, Germany) was measured in the same experiments as TER measurements were performed. 200 μL of LY solution (1 mM in PBS) were applied apically onto the RHE and incubated for 6 h at 37 °C. Subsequently, basal medium was collected and analysed with a Fluorescence reader (Tecan, Männedorf, Switzerland). Permeability was calculated using a standard calibration curve as follows: P_APP_ (cm/s) = flux (µg*s^−1^*cm^−2^)/concentration (µg/ml).

### Biotinylation assay

Human skin samples were injected dermally with 40 µL of 5 mg EZ-Link™ Sulfo-NHS-LC-Biotin (“Biotin-556”; Thermo Scientific, Rockford, USA) in 1 mL PBS with CaCl_2_ directly after excision. Subsequently biopsies were incubated for 2 h on gauze with DMEM, 2% FCS and Pen/Strep (Biochrom AG, Berlin, Germany) at 37 °C. RHE were incubated with 200 μL biotin-solution from the basal side of the model for 30 min (37 °C). Three different biotin-tracers were used: Biotin-556 (0.5 mg/mL), Biotin-dPEG-(24)-NHS (1 mg/mL) (“Biotin-1500”; Iris Biotech, Marktredwitz, Germany), and Biotin-PEG-NHS-5000 (1 mg/mL) (“Biotin-5000”; Santa Cruz Biotechnology, Dallas, USA) in PBS with 1 mM CaCl_2_. Skin samples and RHE were subsequently fixed in 4% Formafix solution (Grimm, Torgelow, Germany) overnight at RT (skin) or up to 48 hours at 4 °C (RHE) and paraffinated.

### Immunofluorescence staining

Immunofluorescence staining of TJ and SC proteins as well as biotin-tracers was performed essentially as described in^21^. For details of staining procedures and evaluation see Supplementary data including Supplementary Fig. S1.

### Electron microscopy

Lanthanum assay was adapted from the method previously described^32^.

#### Evaluation of lipid lamellar organization in RHE

The method which evaluates the proportion of the intercellular space (ICS) occupied by intercellular lipid lamellae (ICLL) was performed as described in^23^. Briefly, the RHE samples were fixed over night at 4 °C in 2% paraformaldehyde and 2.5% glutaraldehyde with 0.1 M cacodylate buffer. After washing in cacodylate buffer (0.4 M) for 1 h, and postfixation with 1% OsO_4_ for 90 min at RT, the samples were shortly washed in distilled water. Dehydration was carried out in an ascending ethanol/propylene oxide series and samples were embedded in Epon. Ultrathin sections were analysed using a TEM CM 10 (FEI Eindhoven, Netherland).

In the TEM images of the SC of RHE a minimum of 5 areas were chosen between corneocytes at different depth. Within the chosen areas in the SC an area of the ICS as well as all the ICLL in this selected area were semiautomatically marked using a software plug-in developed for Image J software (http://rsbweb.nih.gov) and subsequently length of ICLL per ICS was measured. In order to compare ICLL in the different samples, the ratio ICLL/ICS was normalized to an area of 1,000 nm^2^. The normalized ICLLs (nICLL) were used for subsequent statistical analysis^23^.

### Bacterial inoculation

Culture and inoculation with bacteria were performed as previously described^21^. RHE models were infected with 5 × 10^4^ bacteria/cm² in 0.9% NaCl at day 2 and 6 after lifting to air-liquid-interface and incubated for 48 h.

### Western blot and quantitative rt-PCR

Western blot analyses were performed as previously described^21^. For quantitative rt-PCR analyses, total mRNA was isolated from RHE by using the RNeasy mini kit (Qiagen, Hilden, Germany) after homogenisation (3 min, 30 Hz) with sterile grinding balls in the Tissue Lyser (Qiagen) according to the manufacturer’s instructions. First-strand cDNA synthesis (with 0.5 µg RNA template) and real-time PCRs were performed as described^33^.

### Statistical analyses

Data are expressed as mean + SEM. Statistical analyses were performed with analysis of variance (ANOVA) and subsequent Post-Hoc tests with correction for multiple comparisons (Games-Howell (in case of unequal variances), Gabriel’s (in case of similar variances and sample sizes) or Hochberg’s GT2 (in case of similar variances but different sample sizes)) as well as linear mixed models with random intercept fit for the individual experiment. Outliers were excluded according to Extreme Studentized Deviate (ESD) outlier test (n > 15) or to the interquartile range (IQR) with factor 2.2 (n = 1–15)^34,35^. Correlations were calculated by Pearson′s (linear) or Spearman’s (non-linear) rank correlation. For correlations calculated in RHE all siRNA-treated samples (including siRNA control) but not wild type (wt) samples were used to guarantee identical influences except for Cldn-1 levels. Statistical calculations were performed by SPSS 24 (IBM, Armonk, USA). * =  p < 0.05, ** =  p < 0.01; *** = p < 0.001 (same for symbols #, $, §).

## Supplementary information

Dataset 1.

## Data Availability

The datasets generated during and/or analysed during the current study are available from the corresponding author on reasonable request.
