# Supplementary material for: Claudin-1 decrease impacts epidermal barrier function in atopic dermatitis lesions dose-dependently
Source: Sci Rep. 2020 Feb 6;10:2024. doi: 10.1038/s41598-020-58718-9 (PMC7004991; doi:10.1038/s41598-020-58718-9)
Supplement: Supplementary file 1 — Dataset 1. [file 41598_2020_58718_MOESM1_ESM.pdf]

Supplementary data to

Claudin-1 decrease impacts epidermal barrier function in atopic dermatitis lesions dose-dependently

Sophia Bergmann[1], Barbara von Buenau[1], Sabine Vidal-y-Sy[1], Marek Haftek[2], Ewa Wladykowski[1], Pia Houdek[1], Susanne Lezius[3], Hélène Duplan[4], Katja Bäsler[1], Stephan Dähnhardt-Pfeiffer[5], Christian Gorzelanny[1], Stefan W. Schneider[1], Elke Rodriguez[6], Dora Stölzl[6], Stephan Weidinger[6], Johanna M. Brandner [1]

[1] Department of Dermatology and Venerology, University Hospital Hamburg-Eppendorf, Hamburg, Germany

[2] CNRS UMR5305 LBTI and University Lyon1, Lyon, France

[3] Institute for Medical Biometry and Epidemiology, University Hospital Hamburg-Eppendorf, Hamburg, Germany

[4] Pierre Fabre Dermocosmétique, Pierre Fabre Research and Development Center Toulouse, France

[5] Microscopy Services Dähnhardt GmbH, Flintbek, Germany

[6] Department of Dermatology, Venerology and Allergy, University Hospital Schleswig-Holstein, Campus Kiel, Germany

Materials:

Supplementary Table S1: Antibodies and dilutions

Antigen	host species	clone/ labelling	dilution	manufacturer (Cat. No.)
Claudin 1 (Cldn-1)	rabbit	Jay.8	WB 1:1500; IHC 1:500 (skin), 1:700 (RHE)	Invitrogen, Darmstadt, Germany (51-9000)
Claudin 4 (Cldn-4)	mouse IgG1	3E2C1	IHC 1:1000	Invitrogen, Darmstadt, Germany (18-7341)
Occludin (Ocln)	goat	N-19	IHC 1:250	Santa Cruz, Dallas, USA, (sc-8145)
Filaggrin (Flg)	rabbit	polyclonal	IHC 1:2500	Novus Biological, Littleton, USA (NBP1-21310)
Involucrin	mouse IgG1	SY5	IHC 1:1250	Leica, Newcastle, UK (NCL-INV)
Loricrin	rabbit	AF 62	IHC 1:5000	BioLegend, Dedham, USA (PRB-145P)
MIB/Ki67	mouse IgG1, kappa	MIB-1	IHC 1:30	Dako, Glostrup, Denmark (M 7240)
Cytokeratin 10 (CK10)	guinea pig	polyclonal	IHC 1:400	Progen, Heidelberg, Germany (GP-K10)
GAPDH	mouse	GA1R	WB 1:5000	Thermo Fisher, Rockford, USA (MA5-15738)

WB: Western Blot; IHC: Immunohistochemistry; RHE: Reconstructed human epidermis

Supplementary Table S2: FAM dye-labelled real-time PCR TaqMan MGB probes

Molecule	probe labelling	manufacturer
Claudin 1	Hs00221623_m1	All:
Claudin 4	Hs00533616_s1	Applied Biosystems
JAM-A	Hs00170991_m1	(Carlsbad, CA, USA)
Occludin	Hs00170162_m1	
ZO-1	Hs01551861_m1	
Filaggrin	Hs00856927_g1	
Involucrin	Hs00846307_s1	
Loricrin	Hs01894962_s1	
Repetin	Hs03405633_m1	
IL-1beta	Hs01555410_m1	
TLR1	HS00413978_m1	
TLR2	HS01872448_s1	
TLR3	HS01551078_m1	
NOD2	HS01550753_m1	
GAPDH	Hs03929097_g1	

Methods

Genomic analyses of Filaggrin-mutations

Genomic DNA was isolated from peripheral EDTA blood with the automated chemagic Star workstation protocol (Hamilton Company, Reno, NV) and used to determine the filaggrin gene (FLG) status of atopic dermatitis (AD) patients and healthy controls. The four most common European mutations R501X, 2282del4, R2447X and S3247X were analysed with the TaqMan allelic discrimination method (Applied Biosystems, Carlsbad, CA) following the standard procedures based on the manufacturer's reagents. Probe detection was performed with the Applied Biosystems 7900HT Fast Real-Time PCR System.

Immunofluorescence microscopy

Formalin-fixed and paraffinized sections of human skin and reconstructed human epidermis (RHE) were deparaffinized and antigen-retrieval was performed by using 2x10 min microwave heating in TEC buffer (360 W) and incubation with 0.001 % Trypsin (10 min, 37 °C). Next, sections were blocked for 30 min at RT with Dako protein block (Dako, Glostrup, Denmark) and primary antibodies were applied over night at 4°C (for dilutions see Supplementary Table S1). All antibodies but Cldn-1 JAY.8 for skin samples (Dako REAL Antibody Diluent, Dako) were diluted in PBS. After washing, Alexa 488-, Alexa 594- or DyLight 488-coupled secondary antibodies were applied for 30 min at room temperature (RT), followed by another washing and counterstaining with DAPI (1:5000, 1 min at RT). To visualize biotin, Texas red streptavidin (Rockland, Limerick, USA) (1:700 in PBS) was applied together with the secondary antibody onto the biotinylated sections. After a final wash, coverslips were mounted with Flouromount-G (Southern Biotechnology Associates, Inc. Birmingham, AL, USA).

Images were generated using an Axiophot II microscope (Zeiss, Jena, Germany) and Openlab software (version 2.0.9; Improvision, Coventry, UK). All images of a staining series

were taken using the same settings. ImageJ software (version 1.41 n (NIH, Bethesda MD; http://imagej.nih.gov/ij) was used for quantification of Cldn-1, Cldn-4 and Ocln immunostaining-intensity. Evaluation of immunostaining-intensity of Cldn-1 in skin was performed in three layers referred to as "*stratum granulosum* (SG)", "upper *stratum spinosum* (uSSP)", and "lower SSP/*stratum basale* (lSSP/SB)". Therefore, immunostaining intensity of 17 regions of interest (ROIs) per picture were evaluated: 5 ROIs of 137 μm^2 in SG (comprising SG layers 2+3), 5 ROIs of 137 μm^2 in upper SSP (uSSP), 5 ROIs of 320 μm^2 in the area bordering to the basement membrane including *stratum basale* (SB) and lower SSP (lSSP/SB), and two ROIs of 320 μm^2 for background subtraction (Supplementary Fig. S1a). For Cldn-4 and Ocln SG, uSSP and mid SSP (mSSP) which means the SSP directly underneath uSSP were evaluated: 5 ROIs of 137 μm^2 in the SG, 5 ROIs of 137 μm^2 in the uSSP, 5 ROIs of 137 μm^2 in the mSSP, and two ROIs of 320 μm^2 for background subtraction (Supplementary Fig. S1b,c).

Evaluation of "tracer-stops" was performed in AD and healthy controls´ samples by counting these events (arrows in Fig. 2) normalized to the amount of cells in the underlying cell layer visualized by their complete circumference by biotin. In RHE the total number of biotin-stops (arrows in Supplementary Fig. S7 and S8) per visual field (Axiophot II microscope (Zeiss, Göttingen, Germany, 40x objective) was counted. ≥2 (RHE) and ≥3 (skin) visual fields were evaluated in two technical replicates per donor (n = different donors is denoted in the figures).

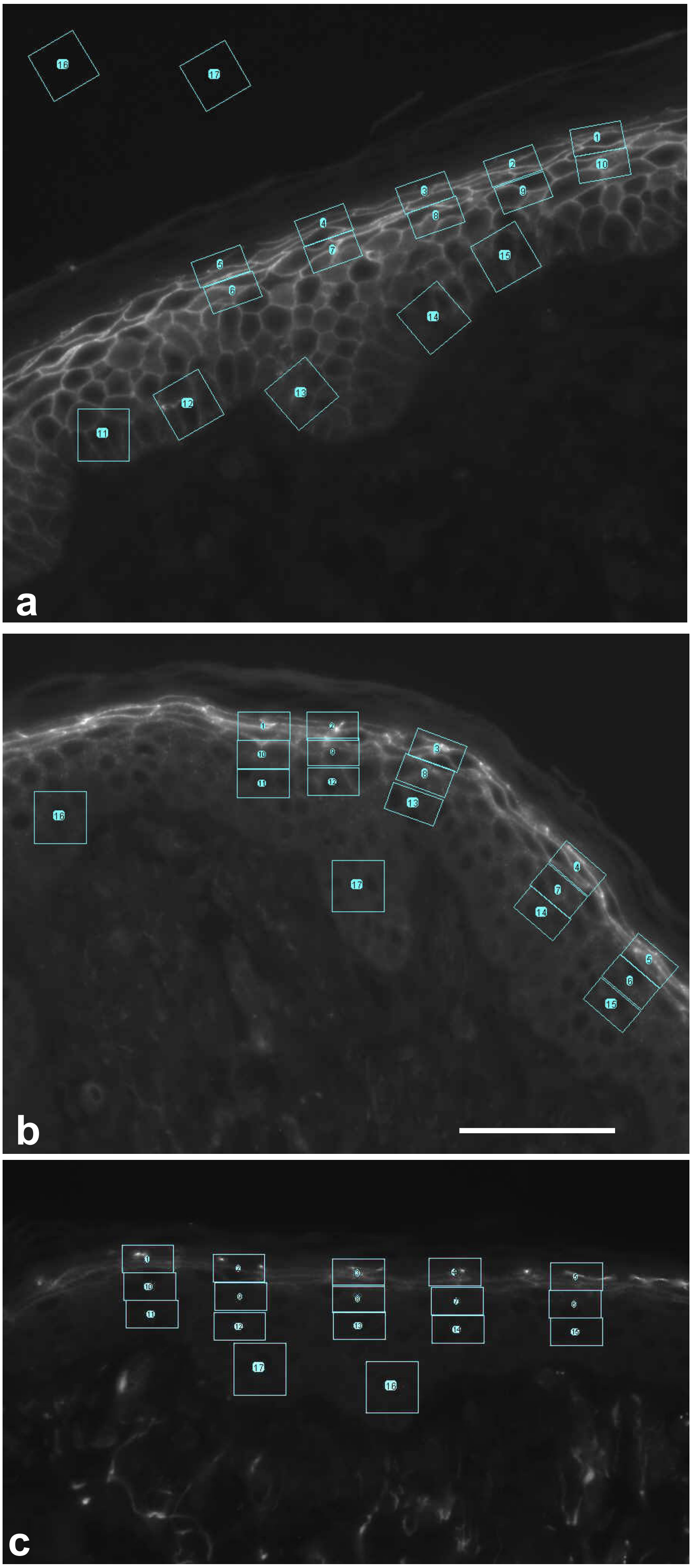

Supplementary Fig. S1. Examples for localization of ROIs to evaluate Cldn-1 (**a**), Cldn-4 (**b**) and Ocln (**c**) immunostaining-intensity in human skin samples. All stainings: ROIs 1-5: SG, ROIs 5-10: uSSP;

Cldn-1: ROIs 11-15: lSSP/SB, ROIs 16, 17: background outside of tissue.

Cldn-4, Ocln: ROIs 11-15: mSSP, ROIs 16, 17: background within the epidermis. ≥3 separate pictures per sample were evaluated and means +/- SEM were calculated. Bar: 50 µm.

Electron microscopy

Lanthanum assay: The RHE samples were fixed with 2 % glutaraldehyde in 0.4 M sodium cacodylate buffer at 4°C. After washes in the same buffer, the basal side of the samples was exposed to post-fixation solution containing 1 % lanthanum nitrate and 1 % OsO_4 in cacodylate buffer for 3 h at room temperature. Next, the samples were rinsed in water, dehydrated in graded ethanol series and embedded in Epon. Ultrathin sections were observed, not counterstained, in a transmission electron microscope equipped with a high resolution digital camera.

Results:

Layer-dependent increase and decrease of Cldn-4 and Ocln immunointensity in lesional and non-lesional AD skin

We observed a trend of upregulation of Cldn-4 in non-lesional compared to healthy skin in all layers. For lesional skin, there was a significant downregulation in SG and a significant upregulation in mSSP (Supplementary Fig. S2). For Ocln, we observed a significant upregulation in SG in non-lesional skin compared to healthy skin and in uSSP and mSSP in lesional skin compared to healthy skin and non-lesional skin (Supplementary Fig. S3).

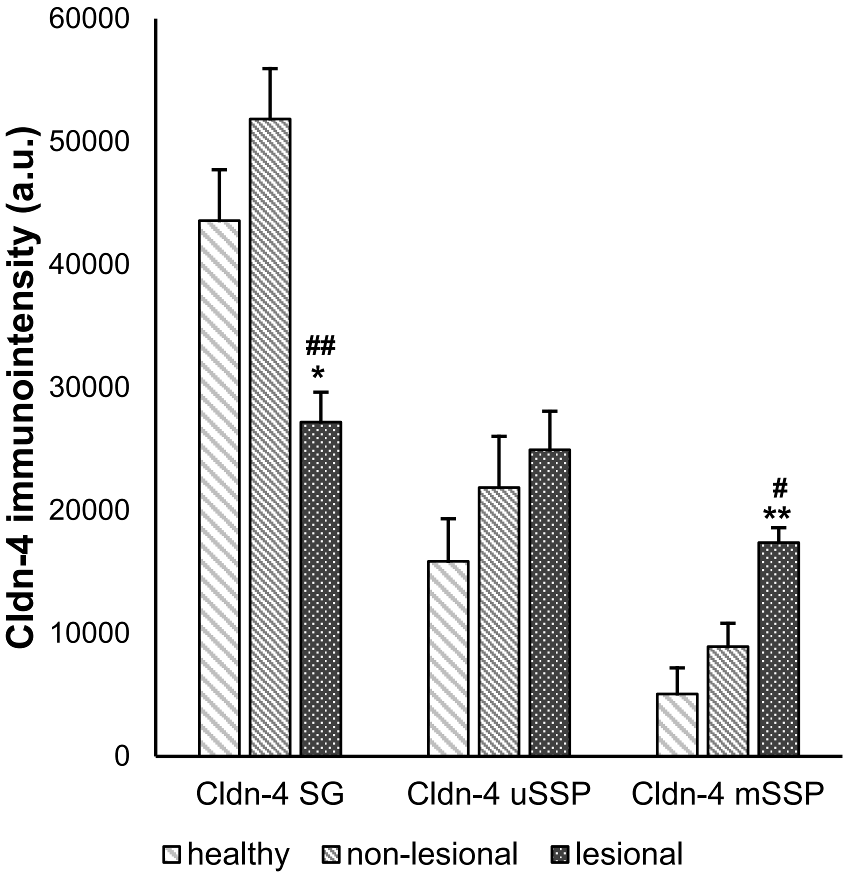

Supplementary Fig. S2. Cldn-4 immunostaining-intensity in healthy, non-lesional and lesional AD samples. SG: *stratum granulosum*; uSSP: upper *stratum spinosum*; mSSP: mid *stratum spinosum*. Mean + SEM, n=13 per group. a.u. arbitrary units; * compared to healthy skin; #: compared to non-lesional skin.

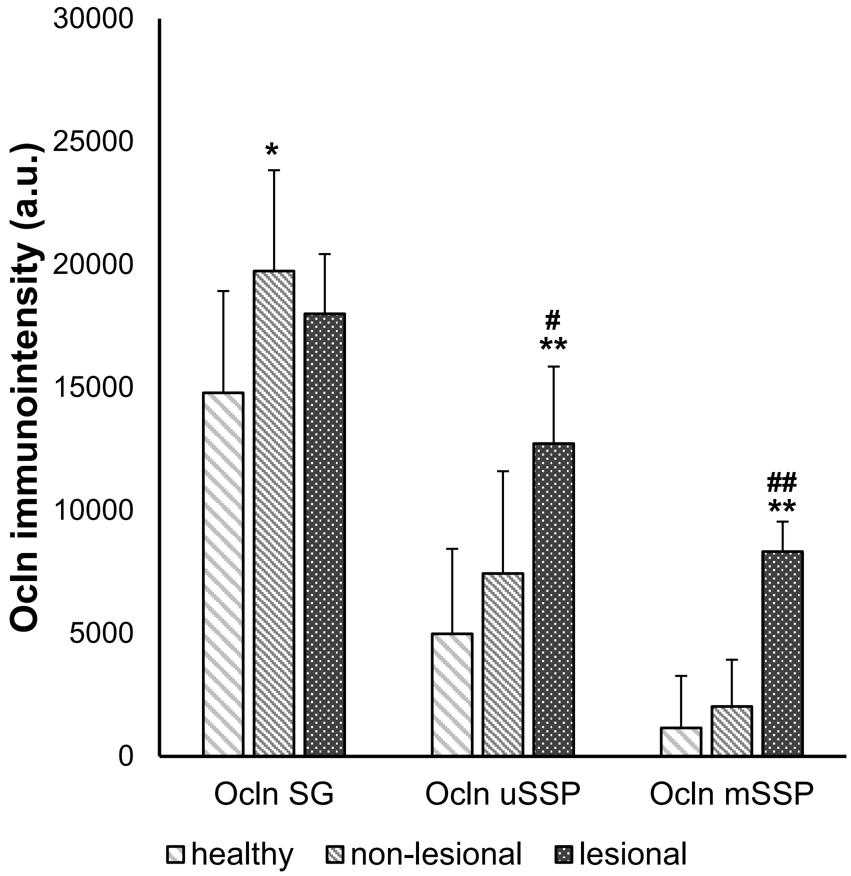

Supplementary Fig. S3. Ocln immunostaining-intensity in healthy, non-lesional and lesional AD samples. SG: *stratum granulosum*; uSSP: upper *stratum spinosum*; SSP: mid *stratum spinosum.* Mean + SEM, n=13 per group. a.u. arbitrary units; * compared to healthy skin; #: compared to non-lesional skin.

Correlation between Biotin-556 tracer stops and Cldn-1 levels in uSSP and lSSP/SB of human skin

There was only moderate correlation between Biotin-556 tracer stops and Cldn-1 levels in uSSP and lSSP/SB (Supplementary Fig. S4).

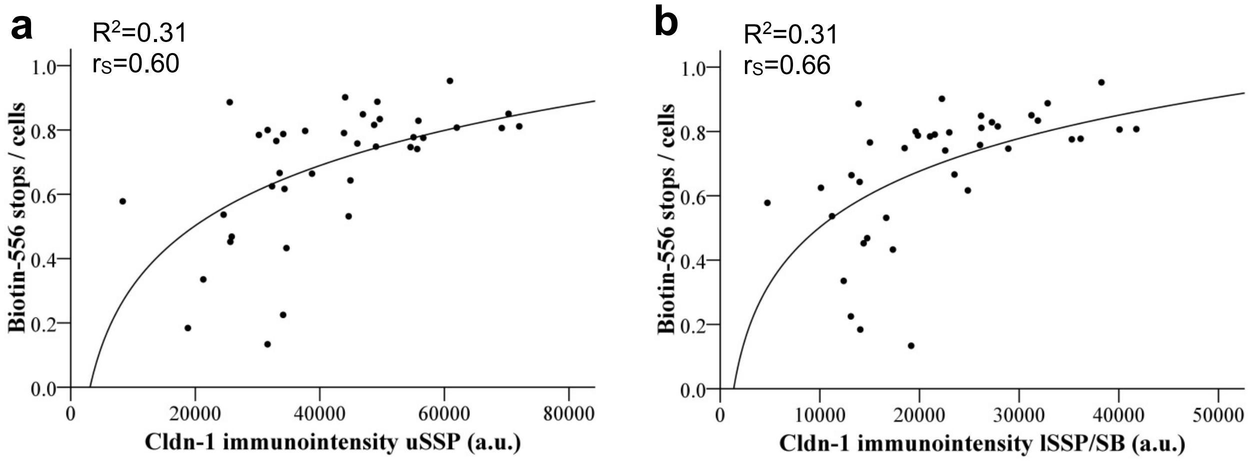

Supplementary Fig. S4. Correlation of Cldn-1 immunostaining-intensity and Biotin-556-tracer stops / cells in upper *stratum spinosum* (uSSP) (**a**) and lower *stratum spinosum* / *stratum basale* (lSSP/SB) (**b**) (n=39). $p<0.01$.

Characterization of reconstructed human epidermis at day 4 and day 8

RHE at day 4 is characterized by 5-7 viable cell layers (comprising 1 layer SB, 3 layers SSP and 1-3 layers SG) and approx. 5 layers of SC (Supplementary Fig. S5). Concerning differentiation markers, cytokeratin 10 was restricted to the suprabasal layers, involucrin was found in the upper SSP and SG, and loricrin and filaggrin were restricted to SG (Supplementary Fig. S5), showing that also at this early time point epidermal differentiation was already distinct. 32.3 ± 5.6 % of all basal cells were proliferative active (Ki67-positive). The RHE were positive for the hyperproliferation marker K6 in the SG and SSP (data not shown). Mean transepithelial electrical resistance (TER) was 1.49 ± 0.1 $kOhm*cm^2$ (n=12), flux of Lucifer Yellow (LY) was $8.14*10^{-8} \pm 5.92*10^{-9}$ cm/s (n=4). Concerning Tight Junction (TJ) barrier function, mean number of biotin-tracer stops / visual field was 4.1 ± 0.8 (n=6) (see also supplementary Fig. S6) for Biotin-556, 5.4 ± 0.3 (n=4) for Biotin-1500 and 4.2 ± 0.7 (n=3) for Biotin-5000.

RHE at day 8 is characterized by 8-10 viable layers (comprising 1 layer SB, approx. 3-5 layers SSP and 3 layers SG) and approx. 12 layers of SC. Localization of differentiation markers was as described for day 4 (Supplementary Fig. S5). 30.7 ± 2.4 % of all basal cells

were proliferative active (Ki-67 positive). K6 was found in the lower SSP only (data not shown). Mean TER was 3.36 ± 0.18 kOhm*cm^2 (n=12), flux of LY 2.24*10^{-9} ± 2.01*10^{-9} cm/s (n=4). Mean number of Biotin-556-tracer stops / visual field was 12.5 ± 1.2 (n=7) (see also supplementary Fig. S6), for Biotin-1500 12.2 ± 0.6 (n=4) and for Biotin-5000 9.8 ± 1.7 (n=4).

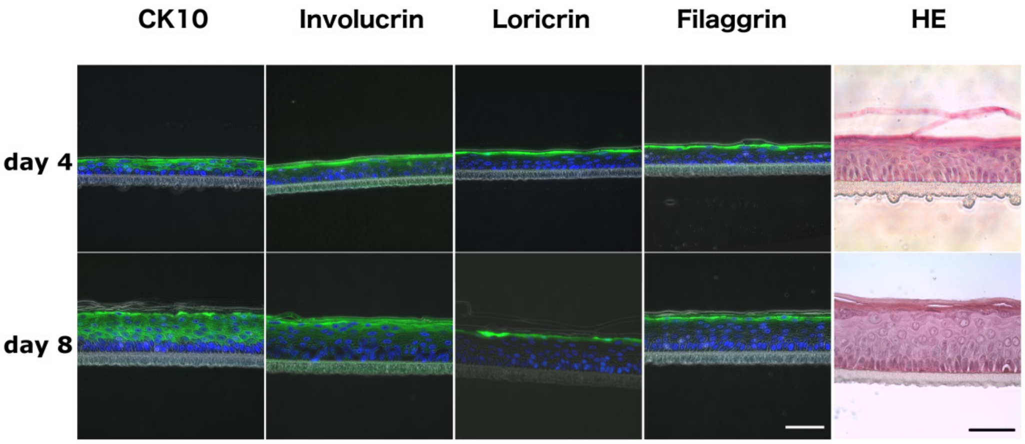

Supplementary Fig. S5. Localization of CK10 (green), involucrin (green), loricrin (green), and filaggrin (green) as well as H+E staining in RHE at days 4 and 8 . Blue: DAPI staining denoting nuclei (day 4 modified from [1]). Bars: 50 µm

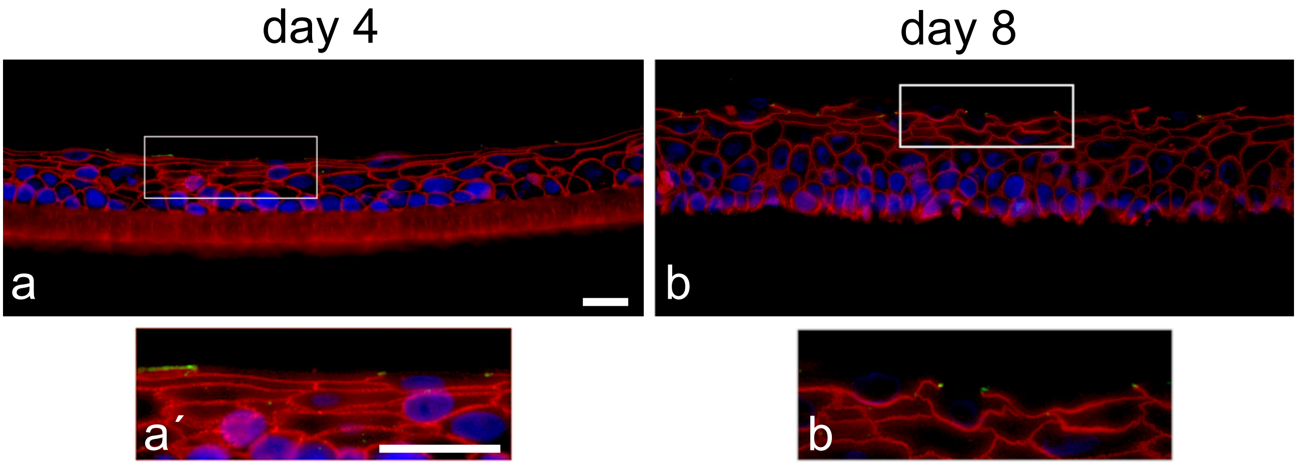

Supplementary Fig. S6. Localization of Biotin-556 (red) and occludin (green) in RHE at day 4 (a, a´) and day 8 (b, b´). (a´,b´) are magnifications of the boxes in (a) and (b). Bars: 20 µm

Knock-down (KD) of Cldn-1 in reconstructed human epidermis at day 4 and day 8

Immunohistochemical stainings of Cldn-1 confirmed that there was a strong decrease of Cldn-1 level after siRNA mediated Cldn-1 KD. Of note, it could be seen that there were

individual cells which still express Cldn-1 while most cells were negative (Supplementary Fig. S7 for day 4 and S8 for day 8). There were no peculiar morphological changes (Supplementary Fig. S7 for day 4 and S8 for day 8), and no significant change in proliferation in KD RHE: day 4: wild-type (wt): 17.6 ± 1.2; siRNA ctrl: 26.5 ± 4.0; No5: 19.1 ± 1.97; No8: 28.2 ± 4.3; $No8_{54}$: 25.3 ± 3.7 Ki67-positive cells / visual field; Day 8: wt: 19.9 ± 4.4; siRNA ctrl: 28.2 ± 4.9; No5: 18.7 ± 3.2; No8: 18.4 ± 1.5; $No8_{54}$: 23.1 ± 2.3. In addition, there was no change of localisation of early (CK10), intermediate (involucrin), and late (loricrin, filaggrin) differentiation markers (data not shown), even though mRNA level of filaggrin decreased at day 8 (see main manuscript).

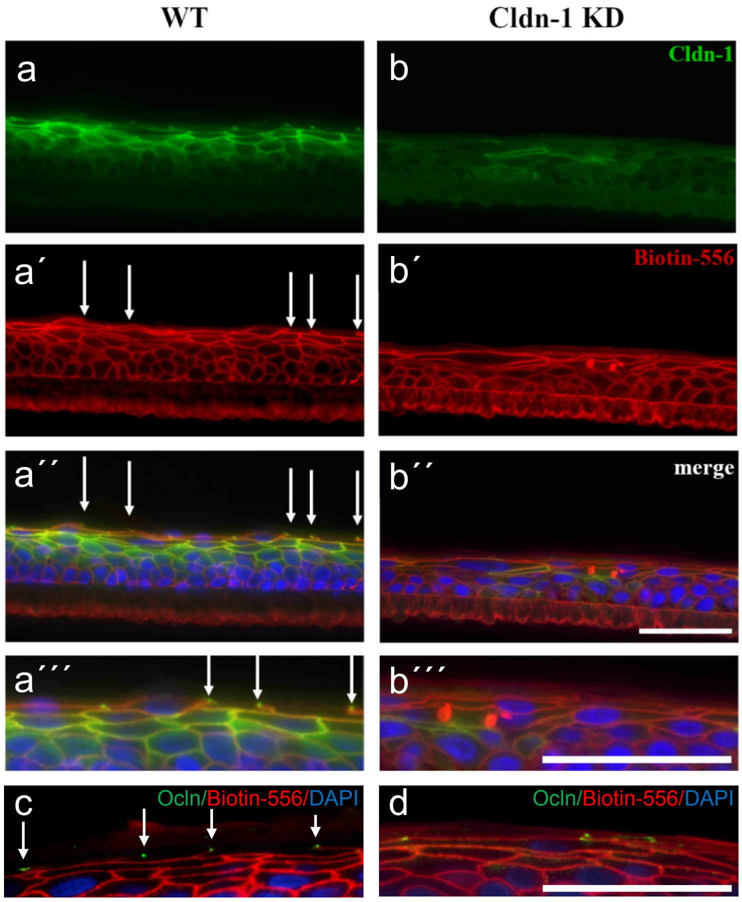

Supplementary Fig. S7. Localization of Cldn-1 (green, **a, a´´, a´´´, b, b´´, b´´´**), Ocln (green, **c, d**) and Biotin-556 (red, **a´, a´´, a´´´, b´, b´´, b´´´, c, d**) at day 4 in WT (**a, a´, a´´, a´´´, c**) and Cldn-1 KD (siRNA No8: **b, b´,b´´, b´´´, d**) RHE. a´´´, b´´´: higher magnification of a´´ and b´´. Blue: DAPI staining denoting nuclei. Arrows denote Biotin-556 tracer stops. Bars: 50 µm.

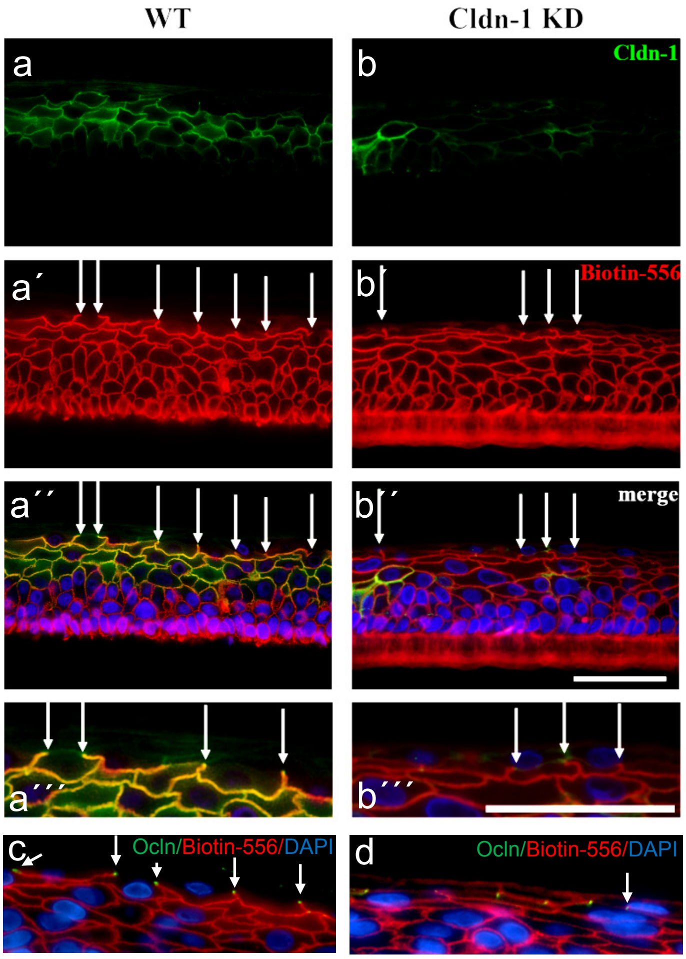

Supplementary Fig. S8. Localization of Cldn-1 (green, **a, a´´, a´´´, b, b´´, b´´´**), Ocln (green, **c, d**) and Biotin-556 (red, **a´, a´´, a´´´, b´, b´´, b´´´, c, d**) at day 8 in WT (**a, a´, a´´, a´´´, c**) and Cldn-1 KD (siRNA No8: **b, b´,b´´, b´´´, d**) RHE. a´´´, b´´´: higher magnification of a´´ and b´´. Blue: DAPI staining denoting nuclei. Arrows denote Biotin-556 tracer stops. Bars: 50 µm.

Effect of Cldn-1 knock-down on barrier function at day 8.

Also at day 8 there was a significant impairment of barrier function to all three biotin-preparations after strong Cldn-1 KD. However, moderate KD as seen with siRNA No5 did not have an effect (Supplementary Fig. S9c,e,g), and siRNA $No8_{58nM}$ had less effect than at day 4. For Biotin-5000 also siRNA $No8_{58nM}$ had no significant effect (Supplementary Fig. S9g). Correlation of biotin-tracer stops with Cldn-1 mRNA levels again fitted significantly to logarithmic curves for Biotin-556 and Biotin-1500 however R^2 values were much lower than at day 4 (Biotin-556: Fig. S9d; R^2= 0.38, r_S= 0.58; p<0.01; Biotin-1500: Fig. S9f; R^2= 0.37; r_S= 0.60; p<0.01). For both tracers a quite stable barrier function at levels >50-60 % was observed,

below this value a clear decrease was seen (Supplementary Fig. S9d,f). For the largest biotin no significant correlation was observed (Supplementary Fig. S9h). For TER and LY permeability, also at day 8 all siRNA treatment groups resulted in dose-dependent significant impairments (Supplementary Fig S9i,k). However, R^2 values were moderately (TER, R^2= 0.32, r_S= 0.57; $p<0.01$), and strongly (LY, R^2= 0.21; r_S= -0.51 $p<0.01$) lower than at day 4. Also at day 8, there was still a dot-like staining of Ocln, but, similar to day 4, often without tracer stop (Supplementary Fig. S8).

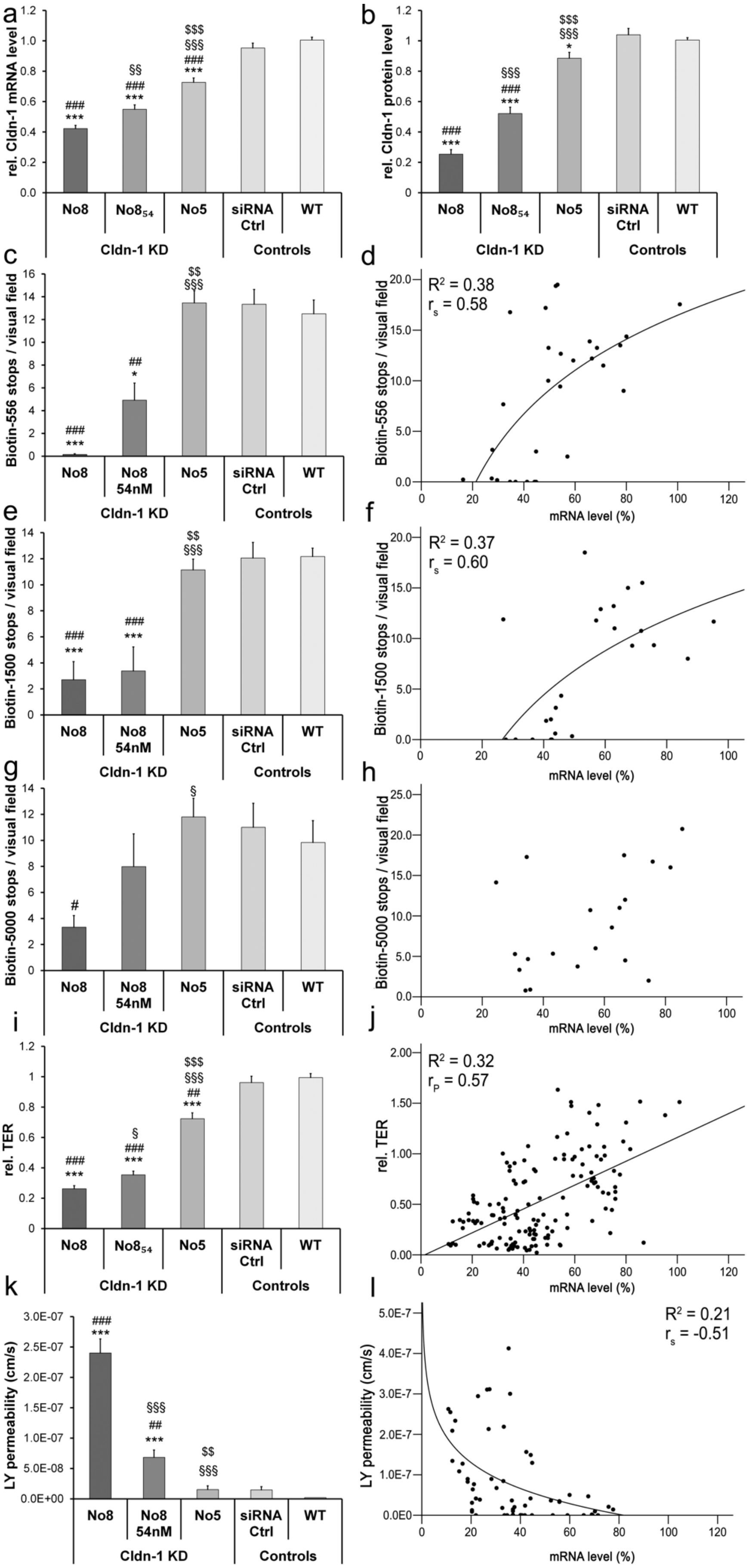
a
rel. Cldn-1 mRNA level
b
rel. Cldn-1 protein level
c
Biotin-556 stops / visual field
d
R² = 0.38
rs = 0.58
e
Biotin-1500 stops / visual field
f
R² = 0.37
rs = 0.60
g
Biotin-5000 stops / visual field
h
i
rel. TER
j
R² = 0.32
rP = 0.57
k
LY permeability (cm/s)
l
R² = 0.21
rs = -0.51
mRNA level (%)
No8
No8₅₄
No8 54nM
No5
siRNA Ctrl
WT
Cldn-1 KD
Controls

Supplementary Fig. S9. Influence of different Cldn-1 levels on TJ barrier and epidermal barrier function in RHE at day 8. (**a, b**) mRNA (**a**) and protein (**b**) levels achieved by the different Cldn-1 knock-down (KD)-siRNA treatments as well as controls normalized to the individual wt control per experiment (a: n=10 different donors, b: n=11 different donors). (**c, e, g, i, k**) Number of Biotin-556 (**c**), Biotin-1500 (**e**), and Biotin-5000 (**g**) stops / visual field, as well as TER (**i**) and Lucifer Yellow (LY) permeability (**k**) achieved by the different Cldn-1 KD-siRNA treatments as well as controls (n= 7, 4, 4, 12, 4 different donors). WT: wild type. (**d, f, h, j, l**) direct correlations of Biotin-556 (**d**), Biotin-1500 (**f**), Biotin-5000 (**h**), TER (**j**) and LY permeability (**l**) to mRNA levels normalized to the wt control with highest Cldn-1 level of all experiments. Significances: * to wt, # to siRNA ctrl, $ to No 8_{54nM} and § to No8.

Cldn-1 knock-down impairs TJ barrier function to lanthanum

We investigated the effect of Cldn-1 KD on lanthanum inside-out penetration. Only the siRNA-preparation inducing strongest KD is illustrated. Cldn-1 KD impairs TJ barrier function to lanthanum (Supplementary Fig. S10).

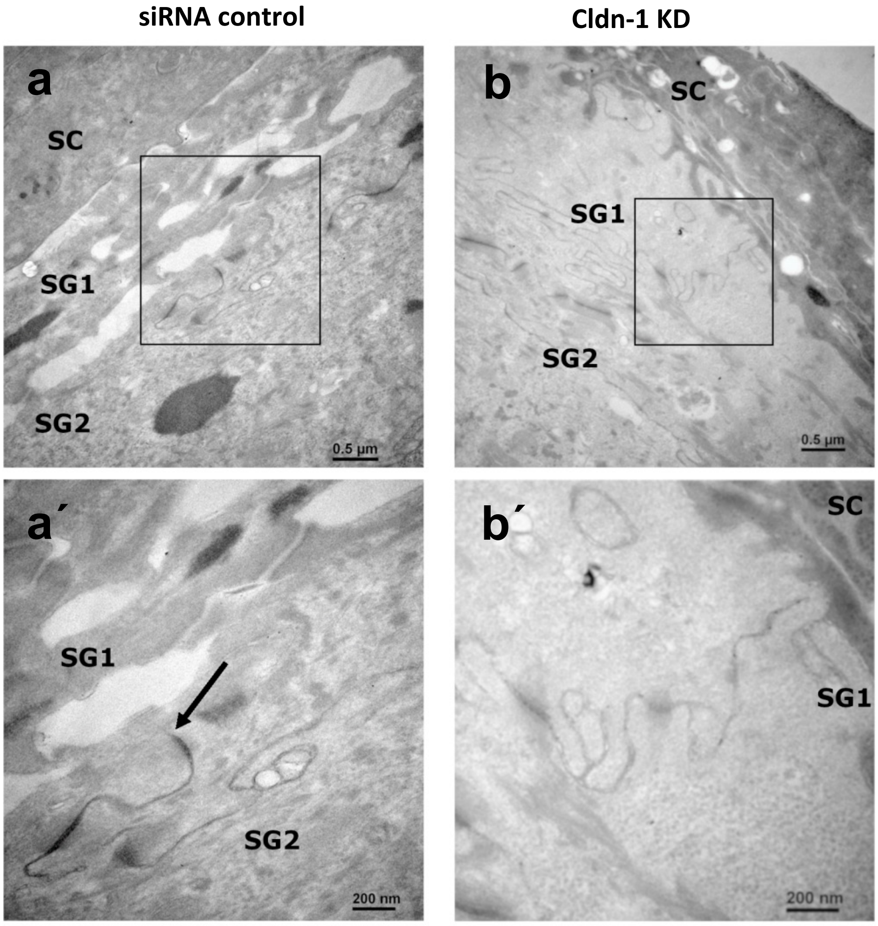

Supplementary Fig. S10. Electron microscopic pictures of lanthanum tracer permeation in RHE at day 4. (**a, a´**) siRNA control, (**b, b´**) Cldn-1 KD (siRNA No8). a´ and b´ are higher magnifications of the boxes in a, b. Arrow in a´ denotes lanthanum tracer stop in SG2. Bars: a, b: 0.5 µm. a´, b´: 200 nm.

No additional effect of staphylococcal infection on barrier function

S. aureus resulted in significant barrier impairment in wt and siRNA control models at day 4 (Supplementary Fig. S11a,b) and by trend at day 8 in siRNA controls (Supplementary Fig. S11c,d). In RHE with strong Cldn-1 KD (No8) decrease of barrier function was substantial and was not intensified by bacterial infection. In RHE with moderate Cldn-1 KD (No 5) a trend of barrier impairing effect by *S. aureus* was seen, but not to a stronger extent as in control models. There was no effect of *S. carnosus* on barrier function (Supplementary Fig. S11).

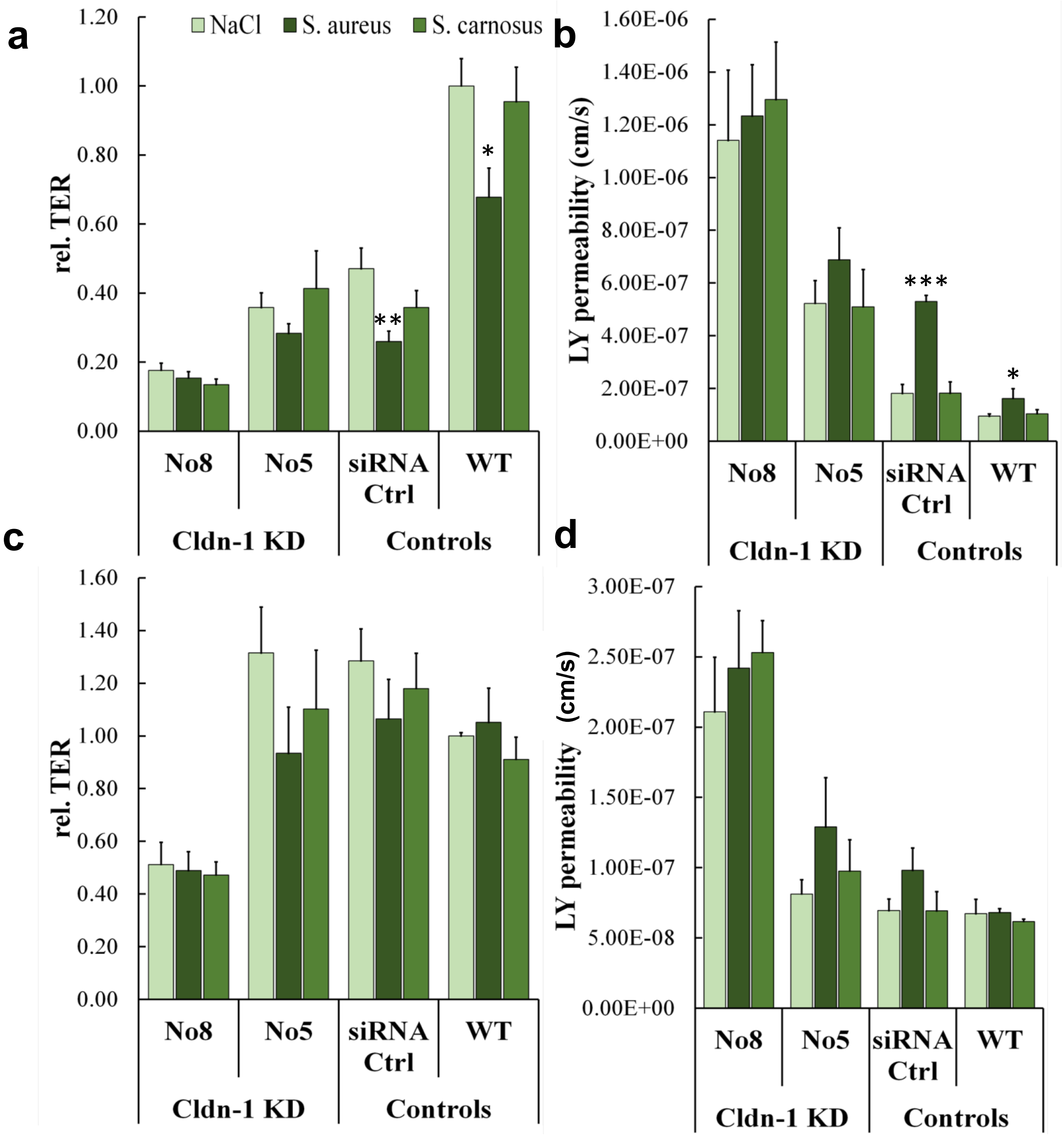

Supplementary Fig. S11. Influence of *S. aureus* and *S. carnosus* infection on barrier function in RHE with and without Cldn-1 KD. (**a, c**) TER; (**b, d**) Lucifer Yellow (LY) permeability. (**a, b**) day 4, (**c, d**) day 8. Mean + SEM. n=3. *compared to NaCl control within each group. Other significances are not marked for better lucidity.

Effect of Cldn-1 knock-down on expression of TJ and SC proteins

For an overview of the effect of knock-down on TJ proteins at day 4 and day 8 and SC proteins at day 8 see Supplementary Tables S3 and S4.

Supplementary Table S3: Influence of Cldn-1 KD on expression of TJ proteins at day 4 and day 8 (fold increase/decrease ± SEM). *** p<0.001 compared to wt, # p<0.05, ## p<0.01 compared to siRNA control. n = 3 different donors in triplicates

	**Day 4**			**Day 8**		
	**siRNA -ctrl**	**siRNA 8**	**siRNA 5**	**siRNA -ctrl**	**siRN A8**	**siRNA 5**
**Cldn-4**	0.91 ± 0.06	0.64 ± 0.05 *** #	0.65 ± 0.03 *** ##	0.87 ± 0.06	1.00 ± 0.06	1.00 ± 0.07
**JAM-A**	1.01 ± 0.02	1.01 ± 0.04	0.92 ± 0.04	1.05 ± 0.03	1.01 ± 0.03	1.12 ± 0.08
**Ocln**	1.38 ± 0.16	1.43 ± 0.08	1.36 ± 0.13	1.10 ± 0.04	1.17 ± 0.12	1.05 ± 0.09
**ZO-1**	1.19 ± 0.09	1.09 ± 0.05	0.87 ± 0.07	1.09 ± 0.05	1.07 ± 0.05	1.08 ± 0.08

Supplementary Table S4: Influence of Cldn-1 KD on expression of SC proteins at day 8 (fold increase/decrease ± SEM). * p<0.05 compared to wt, ## p<0.01 compared to siRNA control. n=4 (Involucrin, Repetin), n=3 (Loricrin, Filaggrin) different donors in triplicates.

	**siRNA-ctrl**	**siRNA8**	**siRNA5**
**Filaggrin**	1.11 ± 0.10	0.68 ± 0.06 * ##	0.88 ± 0.07
**Involucrin**	0.98 ± 0.04	0.91 ± 0.05	0.91 ± 0.05
**Loricrin**	1.01 ± 0.07	0.77 ± 0.07	0.82 ± 0.05
**Repetin**	1.08 ± 0.09	1.45 ± 0.22	2.05 ± 0.39

References

1 Basler, K. *et al.* Biphasic influence of Staphylococcus aureus on human epidermal tight junctions. *Annals of the New York Academy of Sciences* **1405**, 53-70, doi:10.1111/nyas.13418 (2017).
